# Research on the mechanism of Guanyu Zhixie Granule in intervening gastric ulcers in rats based on network pharmacology and multi-omics

**DOI:** 10.3389/fvets.2024.1390473

**Published:** 2024-05-21

**Authors:** Ting Ma, Peng Ji, Fan-Lin Wu, Chen-Chen Li, Jia-Qi Dong, Hao-Chi Yang, Yan-Ming Wei, Yong-Li Hua

**Affiliations:** College of Veterinary Medicine, Gansu Agricultural University, Lanzhou, China

**Keywords:** Guanyu Zhixie Granules, gastric ulcer, network pharmacology, drug interaction, untargeted metabolomics, 16S rRNA

## Abstract

**Objective:**

Guanyu Zhixie Granule (GYZXG) is a traditional Chinese medicine compound with definite efficacy in intervening in gastric ulcers (GUs). However, the effect mechanisms on GU are still unclear. This study aimed to explore its mechanism against GU based on amalgamated strategies.

**Methods:**

The comprehensive chemical characterization of the active compounds of GYZXG was conducted using UHPLC-Q/TOF-MS. Based on these results, key targets and action mechanisms were predicted through network pharmacology. GU was then induced in rats using anhydrous ethanol (1 mL/200 g). The intervention effects of GYZXG on GU were evaluated by measuring the inhibition rate of GU, conducting HE staining, and assessing the levels of *IL-6*, *TNF-α*, *IL-10*, *IL-4*, Pepsin (*PP*), and epidermal growth factor (*EGF*). Real-time quantitative PCR (RT–qPCR) was used to verify the mRNA levels of key targets and pathways. Metabolomics, combined with 16S rRNA sequencing, was used to investigate and confirm the action mechanism of GYZXG on GU. The correlation analysis between differential gut microbiota and differential metabolites was conducted using the spearman method.

**Results:**

For the first time, the results showed that nine active ingredients and sixteen targets were confirmed to intervene in GU when using GYZXG. Compared with the model group, GYZXG was found to increase the ulcer inhibition rate in the GYZXG-M group (*p* < 0.05), reduce the levels of *IL-6*, *TNF-α*, *PP* in gastric tissue, and increase the levels of *IL-10*, *IL-4*, and *EGF*. GYZXG could intervene in GU by regulating serum metabolites such as Glycocholic acid, Epinephrine, Ascorbic acid, and Linoleic acid, and by influencing bile secretion, the *HIF-1* signaling pathway, and adipocyte catabolism. Additionally, GYZXG could intervene in GU by altering the gut microbiota diversity and modulating the relative abundance of *Bacteroidetes*, *Bacteroides*, *Verrucomicrobia*, *Akkermansia*, and *Ruminococcus*. The differential gut microbiota was strongly associated with serum differential metabolites. KEGG enrichment analysis indicated a significant role of the *HIF-1* signaling pathway in GYZXG’s intervention on GU. The changes in metabolites within metabolic pathways and the alterations in *RELA*, *HIF1A*, and *EGF* mRNA levels in RT-qPCR experiments provide further confirmation of this result.

**Conclusion:**

GYZXG can intervene in GU induced by anhydrous ethanol in rats by regulating gut microbiota and metabolic disorders, providing a theoretical basis for its use in GU intervention.

## 1 Introduction

Gastric ulcer (GU) is one of the common peptic ulcer diseases in clinical practice, causing local tissue erosion and necrosis of the gastric mucosa. In severe cases, it can lead to the gastric perforation, characterized by a prolonged course and a high recurrence rate (1). Modern research has identified an association between GU and an imbalance of protective and attack factors in the gastric mucosa, suggesting that assessing these levels can help evaluate the pathological process of GU (2). Recently, with the growth of the breeding and pet industries, there has been an increase in the incidence of GU among livestock, poultry, and pets, including bovine gastritis; chicken gastritis; and GUs in cats and dogs. Currently, the primary treatments for GU in livestock and poultry involve synthetic chemical drugs, which although effective in intervention, have led to adverse reactions and bacterial resistance.

In the theory of traditional Chinese veterinary medicine, GU is classified under “Epigastric Pain,” primarily caused by spleen deficiency and stomach weakness (3, 4). The traditional Chinese medicine compound, Guanyu Zhixie Granules (GYZXG), is based on formulations from “Diyu Tang” and the “Pharmacopoeia of the People’s Republic of China.” It includes *Geranium wilfordii Maxim.*, *Sanguisorba officinalis L.*, *Sophora alopecuroides Linn.*, *Foeniculum vulgare Mill.*, and *Glycyrrhiza uralensis Fisch.* Clinically, it has proven effective in treating GUs in livestock and poultry. The formula primarily uses *Geranium wilfordii Maxim.* and *Sanguisorba officinalis L.*, which help in unblocking meridians, stopping diarrhea and dysentery, cooling blood, detoxifying, and controlling bleeding and healing wounds. *Sophora alopecuroides Linn.* is known for clearing intestines, drying dampness, clearing heat, preventing diarrhea, and resisting ulcers. *Foeniculum Vulgare Mill.* and *Glycyrrhiza uralensis Fisch.* serve as adjuvants to dispel cold, alleviate pain, strengthen the spleen, regulate qi, and harmonize the effects of other medicines (5). The prescription is mild and aims to warm the middle, nourish the spleen, strengthen the body’s foundation, clear heat, dry dampness, and dispel pathogens (5). GYZXG is composed of traditional Chinese medicine herbaceous plants, and its pharmacological active compounds play a role in intervening GU in livestock and poultry. It has the effect of promoting animal growth, and is green and healthy, with low toxic side effects and less susceptibility to drug resistance.

Currently, research on GYZXG is limited to optimizing drug processing and clinical applications. The mechanism of action for its effects on GU remains unclear. Liquid chromatography-tandem mass spectroscopy/mass spectroscopy (LC–MS/MS) can identify and characterize the active compounds in traditional Chinese medicine (TCM), helping to clarify the substances that play significant roles in GU intervention. GU is generally believed to result from external stimuli that damage the gastric mucosa, involving inflammatory factors, cell apoptosis, metabolic pathways, and gut microbiota, among others. Metabolomics, an “omics” approach, mainly studies the chemical processes involving all metabolites in living systems, systematically linking metabolic pathways with disease processes. This provides a comprehensive, holistic new direction for understanding the complex mechanisms and actions of drugs on diseases (6). Moreover, it helps to clarify the intervention mechanisms of GYZXG on anhydrous ethanol-induced rat GUs more effectively. Furthermore, since the stomach and intestines are interconnected, changes in gut microbiota can affect the development of GU. Conducting 16S rRNA gene sequencing on gut microbiota enables a comprehensive analysis to identify specific or group of gut microbiota associated with GU (7). This method can clarify the diversity and composition changes in gut microbiota during the intervention with GYZXG. In addition, network pharmacology analyzes the connections between drugs, targets, and diseases through the construction of a “drug-chemical compound-target-disease” network. This interconnected and systematic approach aligns well with the holistic characteristics of TCM (8).

**Figure 1:**
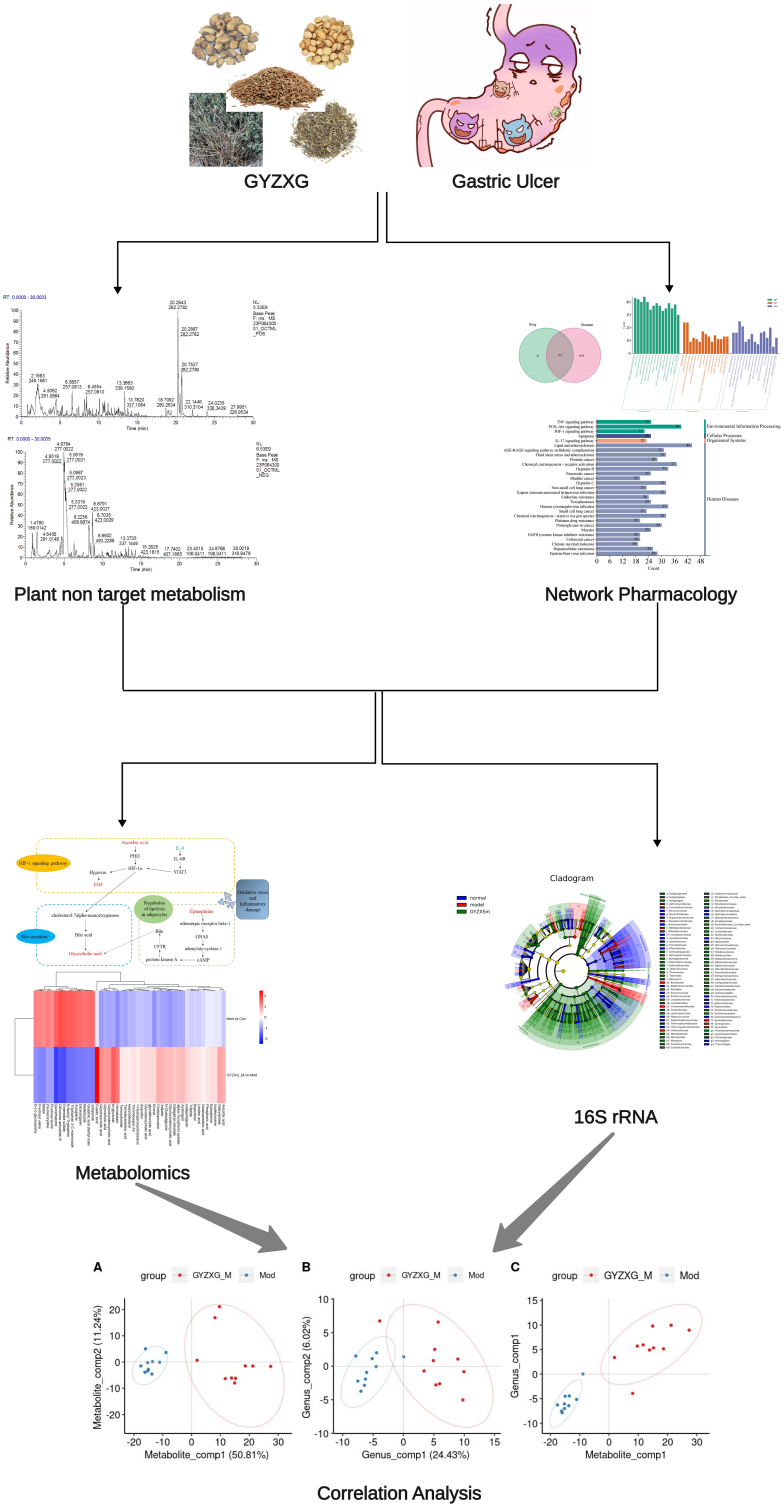
The workflow of this study.

Therefore, to evaluate the protective effects of GYZXG on anhydrous ethanol-induced GU, this study initially identified the main active compounds of GYZXG using UHPLC-Q/TOF-MS technology. Subsequently, a rat model of GU induced by anhydrous ethanol was established to examine the effects of GYZXG. The intervention mechanisms were further explored through studies on gut microbiota, metabolomics, and network pharmacology. These findings provide a theoretical basis for the rational clinical application of GYZXG. The technical route of this study is shown in Figure 1.

## 2 Materials

### 2.1 Experimental animal

Sixty Wistar male rats, each weighing 200 ± 20 g, were provided by the Lanzhou Institute of Veterinary Medicine of Chinese Academy of Agricultural Sciences [No. SCXK (Gan) 2020-0002]. The registration number for the animal experiments is GSAU-Eth-VMC-2019-196, with ethical approval granted on April 12, 2019. All rats were bred at room temperature (23 ± 1°C), with humidity maintained at 50 ± 5%, and exposed to a 12-h light/dark cycle. They had access to a standard diet and water ad libitum. All rats were acclimatized to rearing for 3 days before starting the experiment.

### 2.2 Medicinal materials and experimental reagents

*Geranium wilfordii Maxim.*, *Sanguisorba officinalis L.*, *Sophora alopecuroides Linn.*, *Foeniculum vulgare Mill.*, and *Glycyrrhiza uralensis Fisch.* were provided and identified as qualified by the Gansu Wuwei Tianli pharmaceutical Co., Ltd., Traditional Chinese Medicine Piece Factory. Maltodextrin (Shandong Xiwang Sugar Industry Co., Ltd.). Omeprazole enteric-coated capsules (Shanxi Jinhua Huixing Pharmaceutical Co., Ltd., National Medical Standard: H20045944). Anhydrous ethanol [Tianjin Fuyu Fine Chemical Co., Ltd., (Tianjin) XK 13-011-14001]. *TNF-α*, *IL-10*, *IL-6*, *IL-4*, *PP*, *EGF* (CK-E31063, CK-E30194, CK-E30219, CK-E30217, CK-E30832, CK-E30236, Shanghai Enzyme Biotech Co., Ltd.).

### 2.3 Experimental equipment

The main experimental equipment involved in this experiment includes a swing granulator (Shanxi Tongzheng Biomedical Equipment Design and Manufacturing Co., Ltd., WK-60) and a SpectraMax Plus^384^ microplate reader (Molecular Devices, SpectraMax Plus^384^), etc.

## 3 Methods

### 3.1 Process optimization and preparation of GYZXG

GYZXG was prepared and extracted under the optimal conditions identified by the research team during the preliminary phase (9). Through some methods such as thin-layer analysis, content determination, precision testing, repeatability testing, stability testing, and sample recovery testing, the quality standards for Guanyu Zhixie Granules have been preliminarily established (10). Subsequently, the extract was concentrated, vacuum-dried, mixed with dextrin in a 2:1 ratio, supplemented with 90% anhydrous ethanol as a wetting agent, and finally introduced into the WK-60 rocking granulator to be shaped into particles.

### 3.2 UHPLC-Q/TOF-MS analysis of GYZXG samples

Ultra-high-performance liquid chromatography (Waters 2D UPLC, Waters, United States) in series with a Q-Exactive (Thermo Fisher Scientific, United States) was used to separate and detect metabolites. A Hypersil GOLD aQ chromatography column (100 × 2.1 mm, 1.9 μm, Thermo Fisher Scientific, United States) was employed. The mobile phase consisted of a 0.1% formic acid aqueous solution (liquid A) and 100% acetonitrile containing 0.1% formic acid (liquid B). Primary and secondary mass spectrometry data were collected, with a mass-to-charge ratio range of 150–1,500, primary and secondary resolutions of 70,000 and 35,000, automatic gain control (AGC) settings of 1^e6^ and 2^e5^, maximum injection time (IT) of 100 ms and 50 ms, and fragmentation energy (stepped once) set to 20, 40, and 60 eV. The raw mass spectrometry data (raw file) were imported into Compound Discoverer 3.3 (Thermo Fisher Scientific, United States) for data processing. Metabolites were identified by combining multiple databases such as the BGI Library and mzCloud, and the active compounds of GYZXG were preliminarily identified and characterized with reference to relevant literature.

### 3.3 Network pharmacology analysis

#### 3.3.1 Screening of GYZXG active compounds and corresponding targets

The keywords *Geranium wilfordii Maxim.*, *Sanguisorba officinalis L.*, *Sophora alopecuroides Linn.*, *Foeniculum vulgare Mill.*, and *Glycyrrhiza uralensis Fisch.* from GYZXG were entered into the Traditional Chinese Medicine System Pharmacology Database and Analysis Platform (TCMSP).^1^ The screening criteria were set at Oral Bioavailability (OB) ≥ 30% and Drug Likeness (DL) ≥ 0.18 (11). Active compounds that met the OB and DL thresholds were selected for further analysis. This resulted in the identification of effective active compounds and their corresponding potential action targets.

#### 3.3.2 Screening, analysis, and target gene acquisition of gastric ulcer DEGs

Differential expression analysis data, GSE76588 for GU, were obtained from the GEO database (NCBI).^2^ The species was set to “*Rattus norvegicus*,” and expression profiles from 6 healthy rats and 14 GU rat samples were retrieved. Chip data analysis was conducted using the limma package in R software, which included batch calibration. A filtering threshold for differentially expressed genes (DEGs) was set with a log fold change (FC) > 0.5 and a *p* < 0.05 (12). Heatmaps and volcano plots were generated to visualize the data. Simultaneously, a keyword search for “Gastric Ulcer” was conducted across five major databases: GeneCards,^3^ OMIM,^4^ PharmGkb,^5^ TTD,^6^ and DrugBank.^7^ This was to combine the differential expressed genes from the GEO database with information from other sources to identify GU-related target genes.

#### 3.3.3 Protein–protein interaction network construction and core gene screening

Standardized potential targets and GU target genes from GYZXG were imported into R-4.1.0 software to draw a Venn diagram, aiming to identify potential targets for GYZXG’s intervention in GU. Additionally, the STRING database^8^ was accessed, with the biological species set to “Rat.” The “Multiple protein mode” was selected with a confidence level of ≥0.900, and both the protein–protein interaction (PPI) network diagram and the TSV file were exported for further analysis.

The TSV file was then imported into Cytoscape 3.8.0 for network topology attribute analysis, focusing on the five key topology parameters: Betweenness (BC), Closeness (CC), Degree (DC), Eigenvector (EC), and Local Average Connectivity-based method (LAC), and Network (NC). This analysis helped screen potential targets (13) and construct a functional network of GYZXG’s active compounds acting on GU targets, clarifying the core targets of GYZXG intervention in GU.

#### 3.3.4 GO function and KEGG pathway enrichment analysis

The potential targets identified in section 3.3.3 were imported into R-4.1.0 software and subjected to gene ontology (GO) function and KEGG pathway enrichment analysis using plugins (colorspace, stringi, ggplot2, DOSE, clusterProfiler, and enrichplot). The analysis was performed with a significance threshold of *p* < 0.05 as the screening criteria.

#### 3.3.5 Construction of a “TCM-active compound-target-pathway” network

To reasonably explain the relationship between the active compounds and potential targets of GYZXG, data were imported into Cytoscape 3.8.0 software for visualization and analysis. This process involved examining the network topology structure to determine the degree of connectivity between the active compounds and potential targets. A network diagram was constructed based on their interaction relationships. Active compounds in GYZXG with a connectivity value greater than or equal to the average value were identified as the critical compounds. To reveal the complex relationships among active compounds of GYZXG, GU targets, and signaling pathways, a comprehensive “active compound-target-pathway” network diagram was also analyzed using the same software.

#### 3.3.6 Molecular docking

The key active compounds in GYZXG were selected for molecular docking with the core target of GU using AutoDock Tools 1.5.6 and AutoDock Vina 4.2 software (14). Small molecule ligand structures were obtained from the PubChem database^9^ in mol2 format and refined using ChemOffice software for 3D structural energy minimization. The target protein structures were retrieved from the PDB database^10^ and prepared using PyMOL software, which involved dehydration and, hydrogenation processes. Ligand and receptor files were then converted into PDBQT format using AutoDockTools 1.5.6 to identify and store the locations of their active pockets. Finally, molecular docking was performed by AutoDock Vina 4.2, with results visualized using PyMOL software, displaying binding energies and interaction analyses.

### 3.4 Animal experiments

Sixty Wistar male rats were randomly divided into six groups, with 10 rats in each: a normal control group (received an equal amount of distilled water), a model group, a positive control group (omeprazole enteric-coated capsules, 4.17 mg/kg), a GYZXG low-dose group (GYZXG-L, 0.25 g/mL), a GYZXG medium-dose group (GYZXG-M, 0.5 g/mL), and a GYZXG high-dose group (GYZXG-H, 1 g/mL). The rats were administered their respective treatments by gavage twice a day, 2 mL each time, for 7 consecutive days. On the 8th day, after fasting for 24 h, all groups except the normal control were administered 1 mL/200 g of anhydrous ethanol by gavage to induce GUs (15). One hour later, the rats were anesthetized, and blood was collected. Gastric tissue was harvested and cut along the greater curvature of the stomach. The surface of the gastric mucosa was washed with ice-cold physiological saline, laid flat on a petri dish, and photographed for observation. Parts of the gastric tissue were fixed in 10% paraformaldehyde, and the remainder was stored at −80°C for further testing.

### 3.5 Macroscopic evaluation of gastric ulcers

According to the scoring method of the ulcer index (16, 17), GU injury in rat was evaluated and graded as follows: lesions ≤1 mm were scored as 1 point, >1 mm to 2 mm as 2 points, >2 mm to 3 mm as 3 points, and > 3 mm to 4 mm as 4 points. If the lesion length exceeded 4 mm, it was divided into several segments, with each segment scored according to the above scale. When the lesion width exceeded 2 mm, the score was doubled. The ulcer inhibition rate was calculated using the formula: (model group ulcer index - treatment group ulcer index) / model group ulcer index ×100%.

### 3.6 HE staining and evaluation of cytokines in serum

The gastric tissues of rats from each group were harvested and fixed in paraformaldehyde for 48 h. After undergoing dehydration, embedding and sectioning, the sections were stained with hematoxylin–eosin (HE) and observed under a microscope.

Serum levels of *IL-10*, *IL-6*, *TNF-α*, *IL-4*, *PP*, and *EGF* were detected using ELISA, performed strictly according to the manufacturer’s instructions.

### 3.7 Real-time quantitative PCR

After the stomach tissues had been homogenized in the Scientz-48 L Frozen High-Throughput Tissue Grinder (Scientz, Ningbo, China), an RNA isolater Total RNA Extraction Reagent (Vazyme, Nanjing, China) was used to obtain the total RNA. The reverse transcription process was then carried out using the *Evo M-MLV* RT Mix Kit with gDNA Clean for qPCR Ver.2 (Accurate Biology, Hunan, China). Table 1 shows the primers (Sangon Biotech, Shanghai, China) utilized to amplify cDNA. SYBR Green Premix Pro Taq HS qPCR Kit (Accurate Biology, Hunan, China) and artificial primers for real-time quantitative PCR were combined with the cDNA. Using the 2 − ΔΔCt method, relative gene expression was calculated (18, 19).

**Table 1:** Primer sequences for real-time quantitative PCR.

Gene	Forward primer (5′-3′)	Reverse primer (5′-3′)
*Rela*	CACCTGTTCCAAAGAGCACC	TCTGTGAACACTCCTGGGTC
*Hif1a*	ACCATGCCCCAGATTCAAGA	ATCGCTGTCCACATCAAAGC
*Gapdh*	AACGACCCCTTCATTGACCT	CCCCATTTGATGTTAGCGGG

### 3.8 Serum untargeted metabolomics analysis

#### 3.8.1 Sample collection

Serum samples were collected from three groups—the model, normal control, and GYZXG-M groups (10 cases in each group)—for the untargeted metabolomics analysis.

#### 3.8.2 Data acquisition and processing

The high-resolution mass spectrometer, Q-Exactive, was used in series with a Waters 2D UPLC for metabolite separation and detection. The chromatographic column employed was an ACQUITY UPLC BEH C18 (1.7 μm, 2.1 × 100 mm, Waters, United States). The mobile phase for the positive ion mode consisted of an aqueous solution containing 0.1% formic acid (liquid A) and 100% methanol with 0.1% formic acid (liquid B). For the negative ion mode, it included an aqueous solution with 10 mM ammonium formate (liquid A) and 95% methanol with 10 mM ammonium formate (liquid B). The scan range for the mass-to-charge ratio was 70–1,050, with primary and secondary resolutions of 70,000 and 17,500, AGC settings were 3^e6^ and 1^e5^, with maximum injection times of 100 ms and 50 ms, and fragmentation energy settings of 20, 40, and 60 eV. The raw mass spectrometry data (raw files) were processed using Compound Discoverer 3.1 software (Thermo Fisher Scientific, United States). Principal component analysis was performed on the original multivariate data, and dimensionality reduction was conducted on the observed variables in the dataset. Multivariate statistical analysis [PCA, PLS-DA (20, 21)], univariate analysis (Fold Change, FC), and T-test (Student’s t-test) were used to identify differential metabolites between groups. Furthermore, based on KEGG^11^ and HMDB,^12^ metabolic pathway enrichment analysis was performed on these metabolites. Metabolic pathways with a *p* < 0.05 were significantly enriched in the differential metabolites.

### 3.9 16S rRNA gene sequencing analysis

#### 3.9.1 Sample collection

Intestinal feces were collected and placed in sterile Eppendorf (EP) tubes. They were stored at −80°C and tested by the 16S rRNA sequencing process.

#### 3.9.2 Data acquisition and processing

Microbial DNA was extracted from rat fecal samples using the MagPure Stool DNA KF Kit B, employing 16S rRNA gene v3-v4 primers. The upstream primer was 338F 5′- ACTCCTACGGGGGGCAG-3′, and the downstream primer was 806R 5′- GGACTACHVGGTWTTAAT-3′. The PCR reaction system consisted of 4 μL of 5 × FastPfu Buffer, 2 μL of 2.5 mM dNTPs, 0.8 μL of 5 μM Forward/Reverse Primer, 0.4 μL of FastPfu Polymerase, 30 ng of Template DNA, and ddH_2_O to a total volume of 20 μL. After thoroughly mixing, the reaction mixture was subjected to the following thermal cycling conditions: pre-denaturation at 98°C for 1 min, denaturation at 98°C for 10 s, annealing at 50°C for 30 s, extension at 72°C for 60 s for 30 cycles, and a final extension at 72°C for 10 min. All PCR products were detected by 2% agarose gel electrophoresis, purified with AgencourtAMPure XP magnetic beads, dissolved in the Elution Buffer, labeled, and used for database construction.

The fragment size distribution and concentration of the amplicon library were assessed using an Agilent 2,100 Bioanalyzer. The amplicon library was then paired and sequenced on the HiSeq platform following standard protocols.

### 3.10 Statistical analysis

Data were expressed as mean ± SEM and analyzed using SPSS 26.0 software. Comparisons between groups were performed by a one-way analysis of variance (ANOVA), followed by the least significant difference (LSD) method, with *p* < 0.05 indicating statistically significant differences.

## 4 Results

### 4.1 UHPLC-Q/TOF-MS analysis results of GYZXG samples

**Figure 2:**
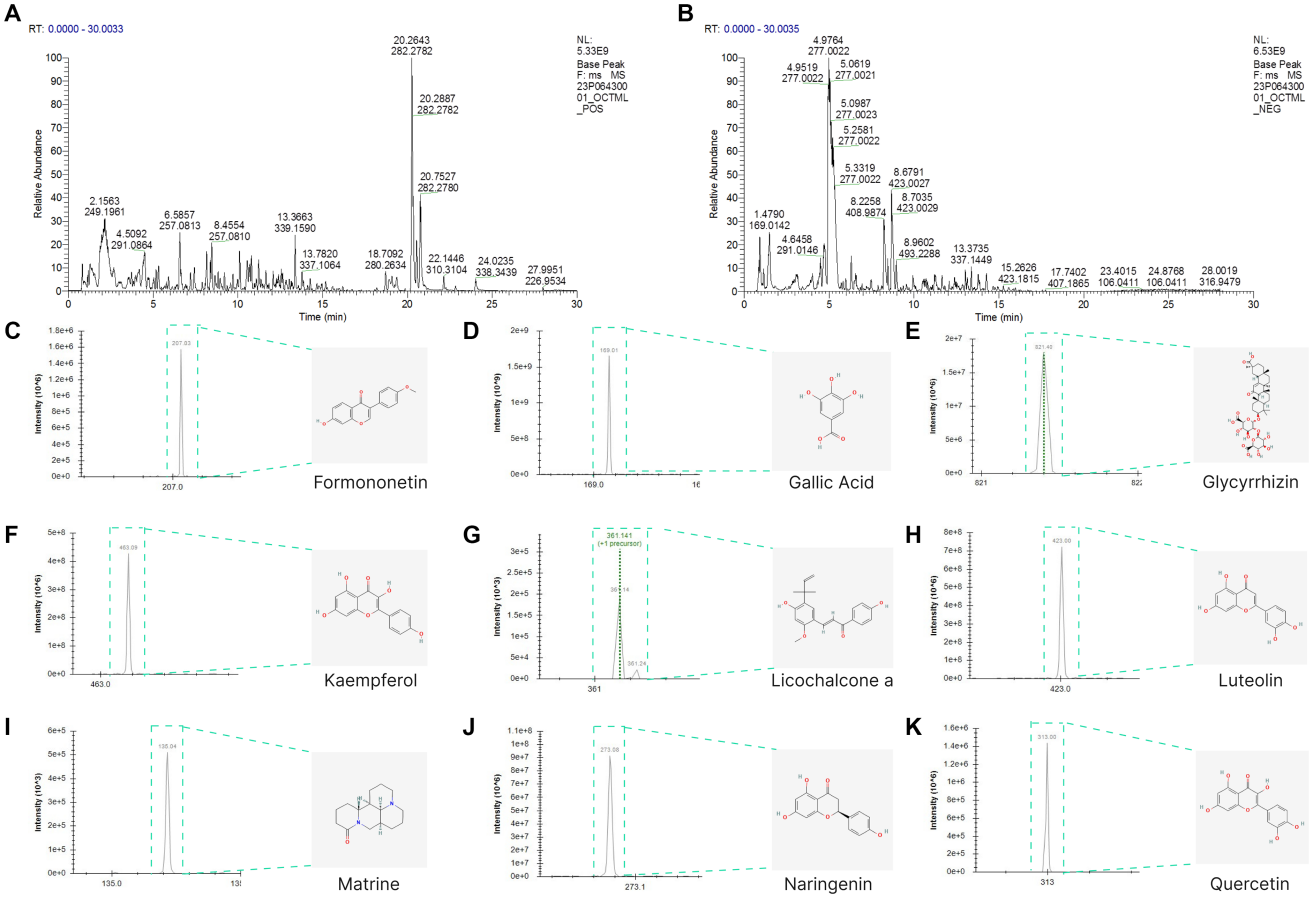
**(A,B)** UHPLC-Q/TOF-MS total ion diagram of Guanyu Zhixie Granules. **(C–K)** Qualitative analysis of 9 active compounds.

The total ion chromatogram, obtained by simultaneously scanning positive and negative ions, showed that the detection peaks of the sample had good shape and large peak capacity (Figures 2A,B). According to the reliability evaluation criteria for metabolite identification, we retained data results for Level 1 and Level 2. Simultaneously, the remaining metabolites (compounds) were compared with the compounds of five drugs in GYZXG, as obtained from the highly reliable scientific database platform, TCMSP,^13^ used in network pharmacology experiments. The qualitative analysis of the compounds was based on retention time, precise relative molecular mass, measurement error, and secondary mass spectrometry fragmentation information from mass spectrometry by a review of the literature. We identified 33 types of compounds including Flavonoids, Triterpenes, Lipids, Tannins, Alkaloids, etc. (Table 2). Nine active compounds were identified according to earlier experimental research on GYZXG (10) and the results of network pharmacology prediction analysis (Table 3; Figures 2C–K).

**Table 2:** Characteristic information and content of compounds in GYZXG.

No	Compound	Source of compounds	Retention/min	Molecular weight	Molecular ion peak[M + H]+	Ppm
1	Glycyrrhizin	*Glycyrrhiza uralensis Fisch*.	11.239	822.40816	821.4012	5.313212854
2	Betulinic acid	*Glycyrrhiza uralensis Fisch*., *Sanguisorba officinalis L*.	9.582	456.36059	385.13	0.541350348
3	Ursolic acid	*Glycyrrhiza uralensis Fisch*.	19.343	456.36027	291.195	−0.161115467
4	Glabrolide	*Glycyrrhiza uralensis Fisch.*	17.25	468.3238	393.35	−0.348692702
5	Narcissoside	*Glycyrrhiza uralensis Fisch.*	7.124	624.16906	311.128	0.035072841
6	Naringin	*Glycyrrhiza uralensis Fisch.*	7.608	580.18056	551.177	2.336747891
7	Rutin	*Glycyrrhiza uralensis Fisch., Geranium wilfordii Maxim.*	6.516	610.15329	563.155	−0.16151486
8	Quercetin 3-o-rhamnoside-7-o-glucoside	*Sanguisorba officinalis L., Glycyrrhiza uralensis Fisch., Sophora alopecuroides Linn., Geranium wilfordii Maxim., Foeniculum Vulgare Mill.*	6.303	610.15323	199.06	−0.261898327
9	Kaempferol-3-o-rutinoside	*Sanguisorba officinalis L., Glycyrrhiza uralensis Fisch., Geranium wilfordii Maxim.*	6.817	594.15827	482.25	−0.330128076
10	Corilagin	*Geranium wilfordii Maxim.*	5.175	634.08302	197.046	3.800186252
11	Isoschaftoside	*Glycyrrhiza uralensis Fisch.*	5.785	564.14818	287.172	0.479869885
12	Schaftoside	*Glycyrrhiza uralensis Fisch.*	5.601	564.14811	411.107	0.35563185
13	Morusin	*Glycyrrhiza uralensis Fisch.*	15.882	420.15718	423.18	−0.254112092
14	Ononin	*Glycyrrhiza uralensis Fisch.*	8.154	430.12642	419.134	0.079061609
15	Liquiritin	*Glycyrrhiza uralensis Fisch.*	6.595	418.1268	477.14	0.998606357
16	Licochalcone c	*Glycyrrhiza uralensis Fisch.*	13.195	338.15189	405.131	0.249264741
17	Licochalcone a	*Glycyrrhiza uralensis Fisch.*	13.374	338.15165	195.138	−0.473997003
18	Isoquercitrin	*Glycyrrhiza uralensis Fisch*., *Geranium wilfordii Maxim.*	6.669	464.09597	197.046	1.058761744
19	Hyperoside	*Geranium wilfordii Maxim.*	5.663	464.09548	271.06	−1.13E-04
20	Astragalin	*Glycyrrhiza uralensis Fisch*., *Geranium wilfordii Maxim.*	7.228	448.10084	153.128	0.632239438
21	Kaempferol-7-o-glucoside	*Sanguisorba officinalis L*., *Glycyrrhiza uralensis Fisch*., *Geranium wilfordii Maxim.*	5.892	448.10045	565.155	−0.253398517
22	Vitexin	*Glycyrrhiza uralensis Fisch.*	6.459	432.10553	193.049	−0.260284976
23	Glabridin	*Glycyrrhiza uralensis Fisch.*	13.858	324.13686	335.218	2.156166367
24	Isobavachalcone	*Glycyrrhiza uralensis Fisch.*	14.606	324.13608	317.173	−0.233833744
25	Glabrone	*Glycyrrhiza uralensis Fisch.*	13.779	336.09902	283.06	−2.239997694
26	Matrine	*Sophora alopecuroides Linn.*	15.643	248.18855	135.0433	−1.243614108
27	Licochalcone a	*Glycyrrhiza uralensis Fisch.*	13.374	338.15165	361.1411	−0.473997003
28	Formononetin	*Glycyrrhiza uralensis Fisch.*	10.800	268.07347	207.0289	−0.316119785
29	Naringenin	*Glycyrrhiza uralensis Fisch.*	9.523	272.06861	273.0759	0.490980209
30	Luteolin	*Geranium wilfordii Maxim.*	8.813	286.04757	423.0029	−0.599842439
31	Quercetin	*Sanguisorba officinalis L., Glycyrrhiza uralensis Fisch., Sophora alopecuroides Linn., Geranium wilfordii Maxim., Foeniculum Vulgare Mill.*	8.77000	302.04257	312.9991	−0.28123648
32	Kaempferol	*Sanguisorba officinalis L., Glycyrrhiza uralensis Fisch., Geranium wilfordii Maxim.*	7.209	286.04769	463.0875	−0.178957882
33	Gallic acid	*Geranium wilfordii Maxim.*	1.493	170.02148	169.0143	−0.2371145

**Table 3:** UHPLC-Q/TOF-MS analysis and identification results.

No.	Compound	Retention/min	Molecular weight	Molecular ion peak[M + H]+	Ppm
1	Matrine	15.643	248.18855	135.0433	−1.243614108
2	Licochalcone a	13.374	338.15165	361.1411	−0.473997003
3	Formononetin	10.8	268.07347	207.0289	−0.316119785
4	Naringenin	9.523	272.06861	273.0759	0.490980209
5	Luteolin	8.813	286.04757	423.0029	−0.599842439
6	Quercetin	8.77	302.04257	312.9991	−0.28123648
7	Kaempferol	7.209	286.04769	463.0875	−0.178957882
8	Gallic acid	1.493	170.02148	169.0143	−0.2371145
9	Glycyrrhizin	11.239	822.40816	821.4012	5.313212854

### 4.2 Network pharmacological analysis results of GYZXG

#### 4.2.1 Potential targets of GYZXG for the intervention on GU

By extracting the active compounds of GYZXG from the TCMSP database, we obtained 13 active compounds from *Sanguisorba officinalis L.* (Diyu), 92 active compounds from *Glycyrrhiza uralensis Fisch.* (Gancao), 14 active compounds from *Sophora alopecuroides Linn.* (Kudoucao), 7 active compounds from *Geranium wilfordii Maxim.* (Laoguancao), and 3 active compounds from *Foeniculum vulgare Mill.* (Xiaohuixiang). Correspondingly, the TCMSP database revealed numerous targets: 344 for *Sanguisorba officinalis L.*, 2,506 for *Glycyrrhiza uralensis Fisch.*, 963 for *Sophora alopecuroides Linn.*, 554 for *Geranium wilfordii Maxim.*, and 611 for *Foeniculum vulgare Mill.* After removing duplicate targets and standardizing protein names via UniProt, we identified 232 potential targets.

**Figure 3:**
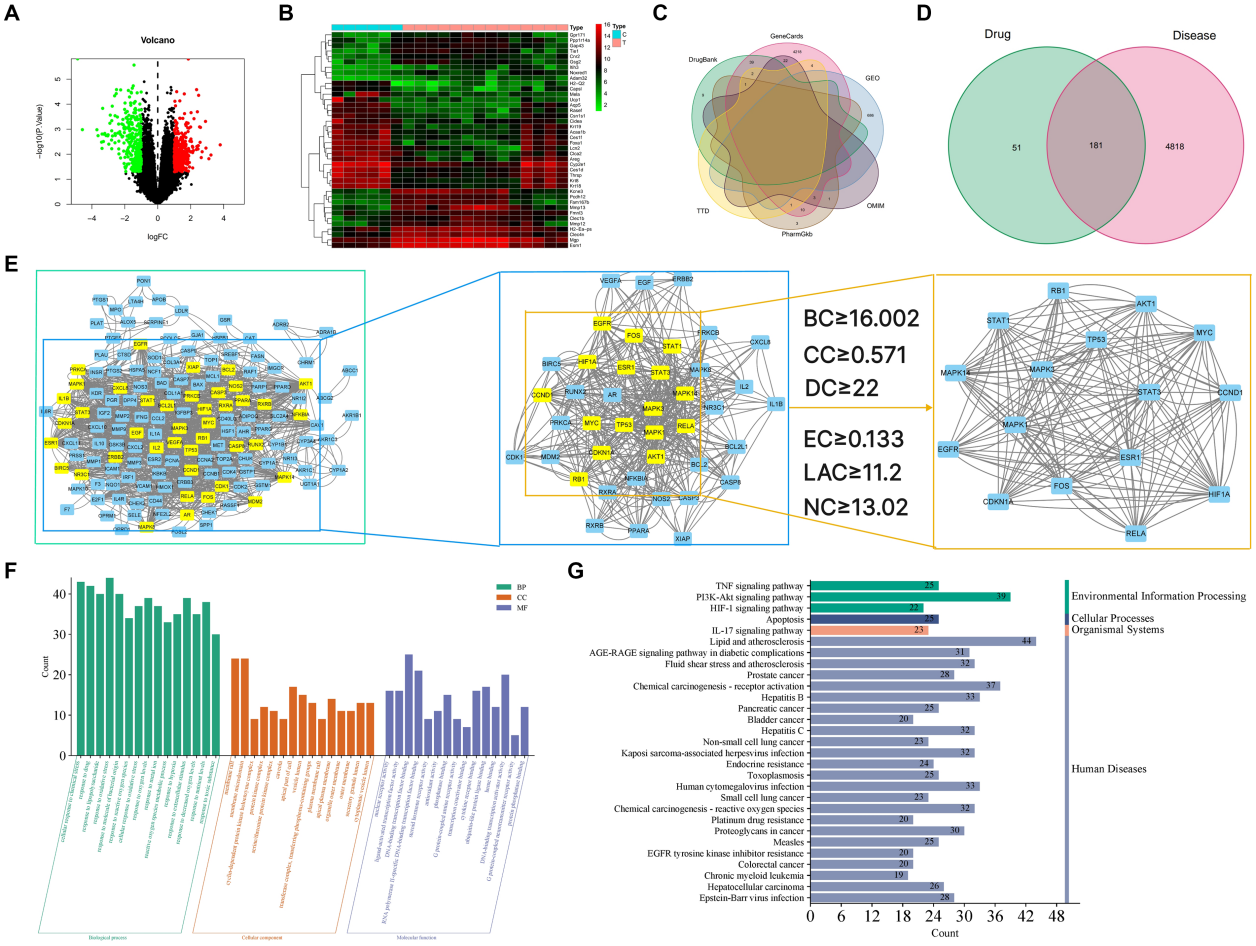
**(A)** Volcano map of differential gene expression. Upregulated genes are indicated in red, downregulated genes are indicated in green, and black represents genes with no significant changes. **(B)** Gene heat map. Upregulated genes are indicated in red (logFC >0) in the genome, downregulated genes are indicated in green (logFC <0) in the genome, and black represents genes that do not have significant differences. The first 6 samples came from the control group, and the last 14 samples came from the disease group. **(C)** Venn diagram of the intersection gene of GU. **(D)** Venn diagram of GYZXG-related targets and GU-related targets. **(E)** Topological analysis of the PPI network. (E1) Interactive PPI network of GYZXG-related targets and GU-related targets. (E2) PPI network of significant proteins extracted from E1. (E3) PPI network of candidate GYZXG targets for GU treatment extracted from E2. **(F)** GO biological function enrichment of GYZXG in the treatment of GU. **(G)** KEGG signaling pathway enrichment of GYZXG in the treatment of GU.

Gene data of GU rats were obtained from the GEO database. We compared gene expression levels between the GU experimental group and the normal group, identifying differentially expressed genes (DEGs). From the GSE76588 gene chip analysis, 686 genes were noted in GU rat experimental samples, with 317 upregulated and 369 downregulated (Figure 3A). These 686 differentially expressed genes between the experimental and normal group were visualized in the form of heatmaps (Figure 3B). Additionally, by querying GeneCards, OMIM, PharmGkb, TTD, and DrugBank databases, and combining this with GEO DEGs, we obtained 4,999 GU-related genes in rats. Duplicate targets were removed for Venn diagram analysis (Figure 3C).

#### 4.2.2 PPI network analysis results

Venn diagrams were plotted by overlapping the potential action targets of GYZXG with rat GU target genes, resulting in 181 common action targets (Figure 3D). These common targets were uploaded to the STRING platform to obtain PPI data, and the network was subsequently constructed and visualized using Cytoscape 3.8.0. As shown in Figure 3E, the PPI network comprised 152 nodes and 1,352 edges. Multiple scoring and filtering operations were conducted using the CytoNCA plugin, employing median values as screening criteria: BC of 16.002, CC of 0.571, DC of 22, EC of 0.133, LAC of 11.2, and NC of 13.02. This analysis resulted in the identification of 16 core genes (Table 4), which may play a vital role in influencing the GYZXG intervention on GU through the PPI network.

**Table 4:** Detailed information on the 16 core genes obtained through screening.

No.	Gene name	Degree
1	*RELA*	42
2	*ESR1*	34
3	*AKT1*	42
4	*MAPK14*	38
5	*MAPK3*	54
6	*STAT3*	50
7	*TP53*	46
8	*STAT1*	26
9	*MAPK1*	52
10	*FOS*	32
11	*EGFR*	28
12	*CCND1*	26
13	*RB1*	26
14	*MYC*	34
15	*HIF1A*	30
16	*CDKN1A*	28

#### 4.2.3 Enrichment results of GO and KEGG pathways in GU by GYZXG

To further investigate the mechanism of action of GYZXG against GU, GO function and KEGG pathway enrichment analyses were completed. These analyses identified the biological characteristics of the common targets between GYZXG and GU in detail. According to the screening criteria of *p* < 0.05, 2,678 GO entries were identified. The top 15 enriched entries in biological process (BP), cellular composition (CC), and molecular function (MF) were visualized (Figure 3F). KEGG pathway analysis showed that a total of 175 pathways are involved in the intervention on GU by GYZXG. Figure 3G displays the top 30 significantly enriched pathways, clearly indicating that the intervention on GU by GYZXG is primarily related to inflammation-related pathways.

#### 4.2.4 Construct “TCM-active ingredient-target-pathway” network relationship diagram

**Figure 4:**
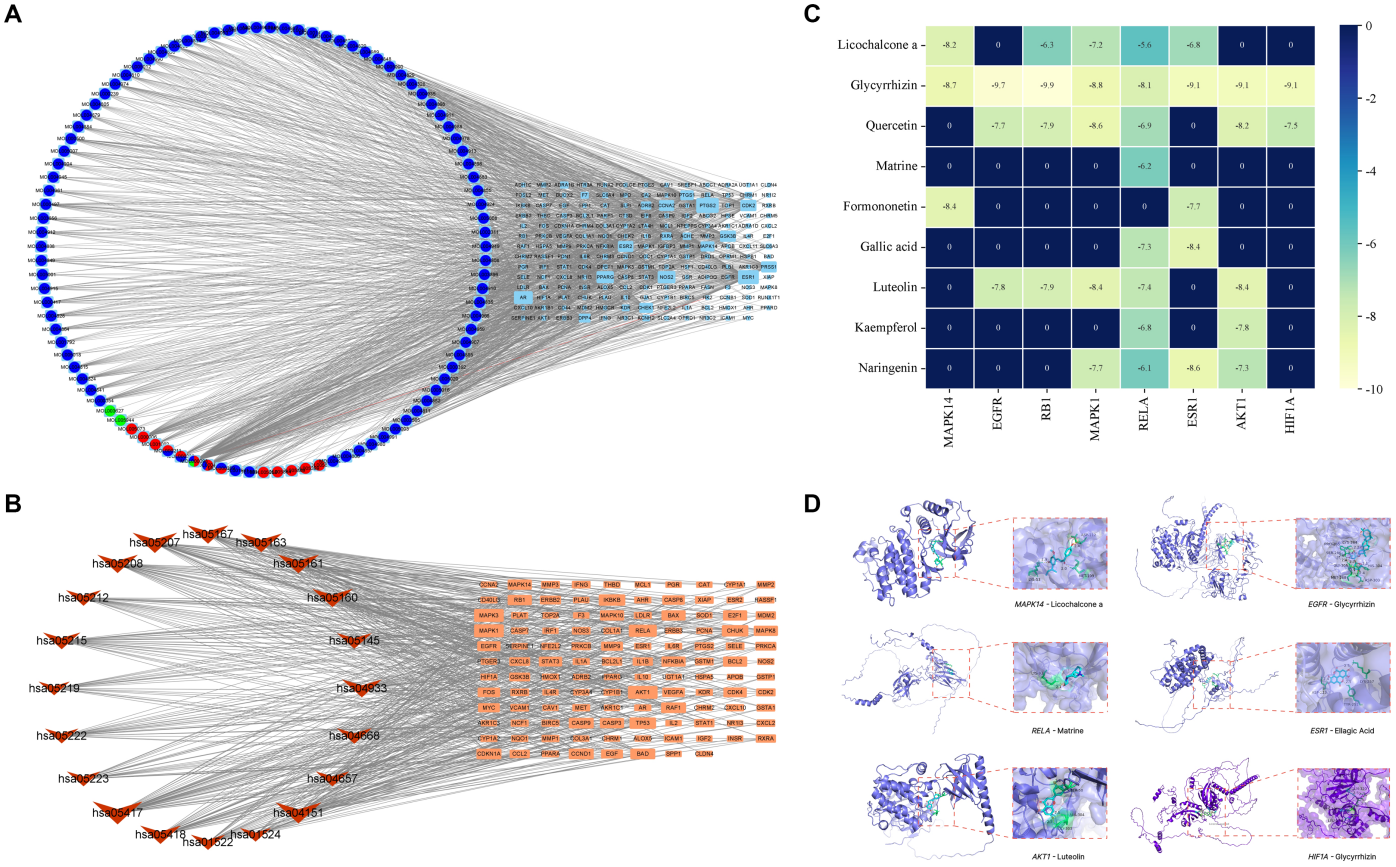
**(A)** Compound-target network of GYZXG in intervening GU. Light blue, green, light red, dark blue and dark red circles represent the compounds from Gancao, Kudoucao, Laoguancao, Xiaohuixiang and Diyu; circle nodes of more than two colors are multiple drug compounds; and cyan squares represent targets. **(B)** The target signal pathway network is represented by red inverted triangle nodes representing the path and orange square nodes representing the target. **(C)** Molecular docking binding energy heatmap. **(D)** Docking patterns of key targets and specific active compounds.

Based on the scores from the “cytoNCA” plugin in Cytoscape 3.8.0, along with results from the initial thin-layer chromatography and high-performance liquid chromatography experiments for GYZXG (10), a comprehensive screening was performed on 181 common targets between GYZXG and GU. As a result, 9 core compounds were identified as critical nodes (refer to Figure 4A; Table 5). Regarding pathways, the four with the highest correlation were identified as the *HIF-1* Signaling pathway, the lipid and atherosclerosis signaling pathway, the *PI3K/Akt* signaling pathway, and the *TNF* signaling pathway (Figure 4B).

**Table 5:** Nine main active compounds in GYZXG.

No.	Compound	Degree
1	Quercetin	Experimentation (10)
2	Matrine	Experimentation (10)
3	Formononetin	22
4	Naringenin	27
5	Licochalcone A	26
6	Ellagic acid	Experimentation (10)
7	Luteolin	46
8	Kaempferol	41
9	Glycyrrhizic acid	Experimentation (10)

#### 4.2.5 Molecular docking results

To further verify the effect of GYZXG’s active compounds on rat GU, molecular docking technology was employed. The eight most relevant core targets were selected as receptors and docking was conducted with nine core compounds as ligands. The binding energies were recorded (Figure 4C), with values less than −5 kcal/mol indicating stable binding; lower value suggest a more stable bound conformation. Based on the magnitude of these binding energies, the top six groups with the most stable interactions were selected for further visualization and analysis (Figure 4D; Table 6).

**Table 6:** Detailed information on molecular docking targets and active compounds.

Target	Uniprot ID	Center coordinates	Compounds Mol ID	PubChem ID	Compounds name
*MAPK14*	AF-P70618-F1	2.17, 2.64, −0.255	MOL000497	5,318,998	Licochalcone A
*EGFR*	AF-Q01279-F1	−6.013, 4.751, −10.254	MOL004876	14,982	Glycyrrhizin
*RELA*	AF-Q04207-F1	−1.476, −2.077, −8.489	MOL005944	91,466	Matrine
*ESR1*	AF-P06211-F1	−4.277, −7.388, 4.482	MOL001002	5,281,855	Ellagic acid
*AKT1*	AF-P47196-F1	0.293, 6.819, 4.478	MOL000006	5,280,445	Luteolin
*HIF1A*	AF-O35800-F1	−4.049, 2.042, 1.975	MOL004876	14,982	Glycyrrhizin

### 4.3 Weight changes in GU rats during administration

**Figure 5:**
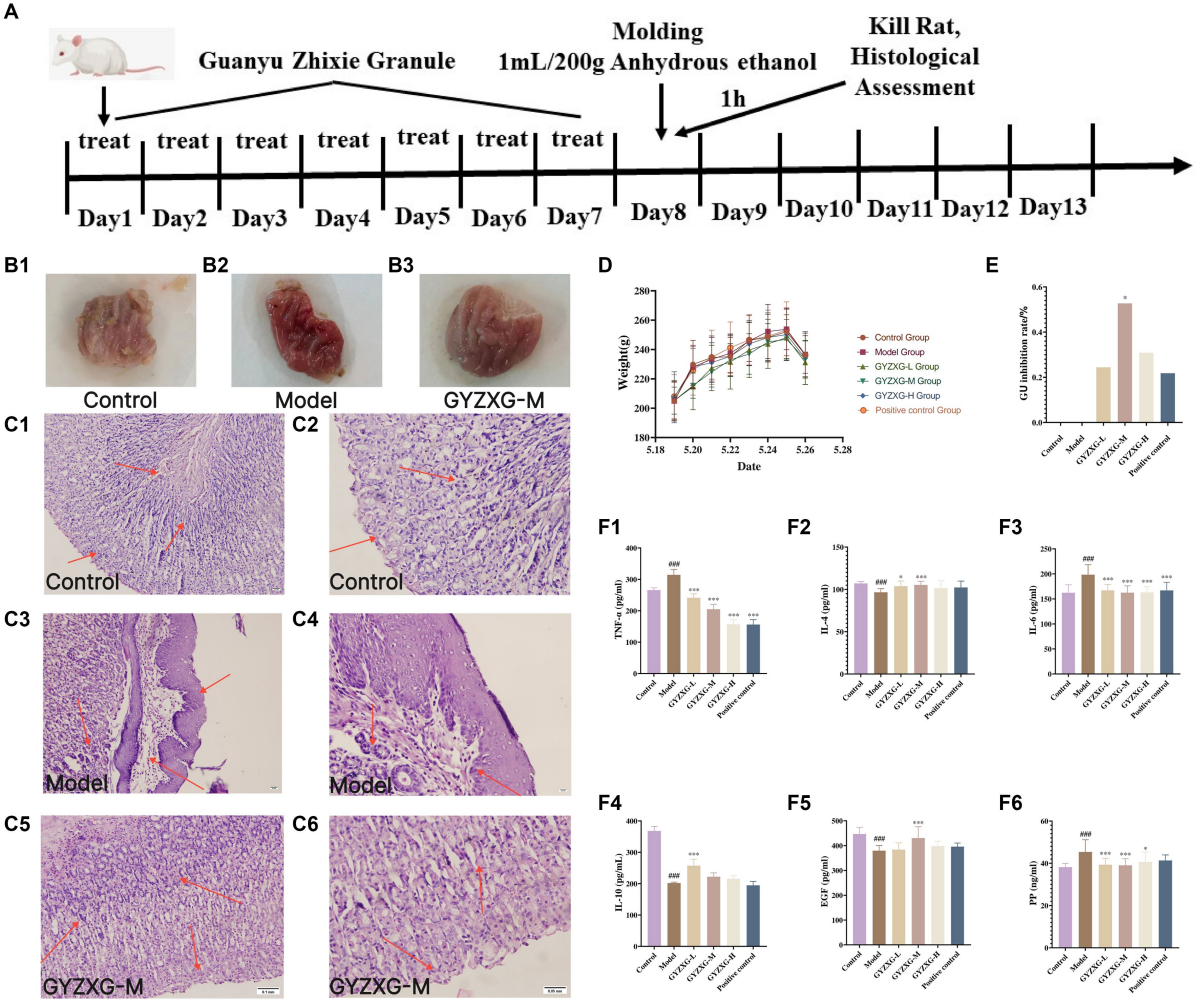
**(A)** GYZXG intervention against anhydrous ethanol-induced GU model in rats. Appearance of the ethanol-induced gastric mucosa in rats: **(B1)** Normal control group; **(B2)** Model group; **(B3)** GYZXG medium-dose group. Effect of GYZXG on the microscopic appearance of the ethanol-induced gastric mucosa in rats (HE staining, ×200 and ×400): **(C1,C2)** Normal control group; **(C3,C4)** Model group; **(C5,C6)** GYZXG medium dose group. **(D)** Body weight changes in rats during the experimental period. **(E)** GU inhibition rate. **(F1–F6)** Changes in the levels of *IL-10*, *IL-4*, *IL-6*, *TNF-α*, *EGF*, and *PP* in the serum of rats.

During the administration period, each group of rats exhibited a significant increase in feed intake and body weight, although no significant differences were observed between the groups (*p* > 0.05). This suggests that GYZXG may have contributed to strengthening the spleen and stomach, boosting feed intake, and enhancing the body’s immunity (Figures 5A,D).

### 4.4 Effect of GYZXG on the inhibition rate of gastric ulcer in various groups of rats

The gastric wall and mucosa of the rats in the normal control group rats were intact, with no damage such as bleeding or ulcers observed. In contrast, the gastric mucosa of the model group rats was damaged; the surface appeared light pink and congested, and mucus was observed oozing from the mucosal surface. Ulcer depressions on the gastric mucosa wall were present, and the folds around the ulcers had disappeared, forming obvious hemorrhagic necrosis and ulcer blood spots, indicating that the GU model was successfully established. Compared with the model group, the degree of gastric mucosal damage in each treatment group was significantly reduced, with considerable decreases in the ulcer surface and reduced congestion. Additionally, the ulcer inhibition rate in the GYZXG-M group was significantly increased (*p* < 0.05; Figures 5B1–B3,E).

### 4.5 Effect of GYZXG on gastric histopathology in various groups of rats

Under the microscope, HE staining revealed that in the normal control group, gastric mucosal epithelial cells were neatly arranged, and the tissue structure of gastric mucosal layer cells was clear, with no inflammatory cell infiltration or edema in the submucosal layer and mucosal lamina propria. In contrast, the model group exhibited shedding and necrosis of upper mucosal cells, along with extensive loss and necrosis of the gastric mucosal layer, submucosal layer, and mucosal lamina propria. There was also notable inflammatory cell exudation, and the gaps between glands widened. Compared with the model group, each administration group showed varying degrees of reduced gastric mucosal injury. Notably, in the GYZXG-M group, GUs were significantly reduced, with fewer hemorrhagic foci and less inflammatory cell infiltration. The damage to the mucous layer and submucosal layer was largely repaired, showing only slight injuries. However, glandular disorders and a small number of inflammatory cells were still observed (Figures 5C1−C6).

### 4.6 Effect of GYZXG on related inflammatory factors in the serum of rats of each group

Compared with the normal control group, the levels of anti-inflammatory factors *IL-10* and *IL-4* in the model group rats’ serum were significantly reduced (*p* < 0.01). In contrast, the pro-inflammatory factors *IL-6* and *TNF-α* levels significantly increased (*p* < 0.01). Compared with the model group, the anti-inflammatory factor *IL-10* was extremely significantly increased in the GYZXG-L group (*p* < 0.01), the anti-inflammatory factor *IL-4* was extremely significantly increased in the GYZXG-M group (*p* < 0.01), and increased dramatically in the GYZXG-L group (*p* < 0.05); The levels of pro-inflammatory factors *IL-6* and *TNF-α* were extremely significantly reduced in each treatment group (*p* < 0.01; Figures 5F1−F4).

Compared with the normal control group, the serum *PP* content in the model group was significantly increased (*p* < 0.01), while the *EGF* content was considerably reduced (*p* < 0.01). In comparison to the model group, serum *PP* levels in the GYZXG-L/M groups were significantly reduced (*p* < 0.01), and in the GYZXG-H group, they were also significantly decreased (*p* < 0.05). The *EGF* content in the GYZXG-M group significantly increased (*p* < 0.01). These results indicate that GYZXG had a strong protective effect against anhydrous ethanol-induced GUs (Figures 5F5,F6).

### 4.7 Validation of the *HIF-1* signaling pathway

**Figure 6:**
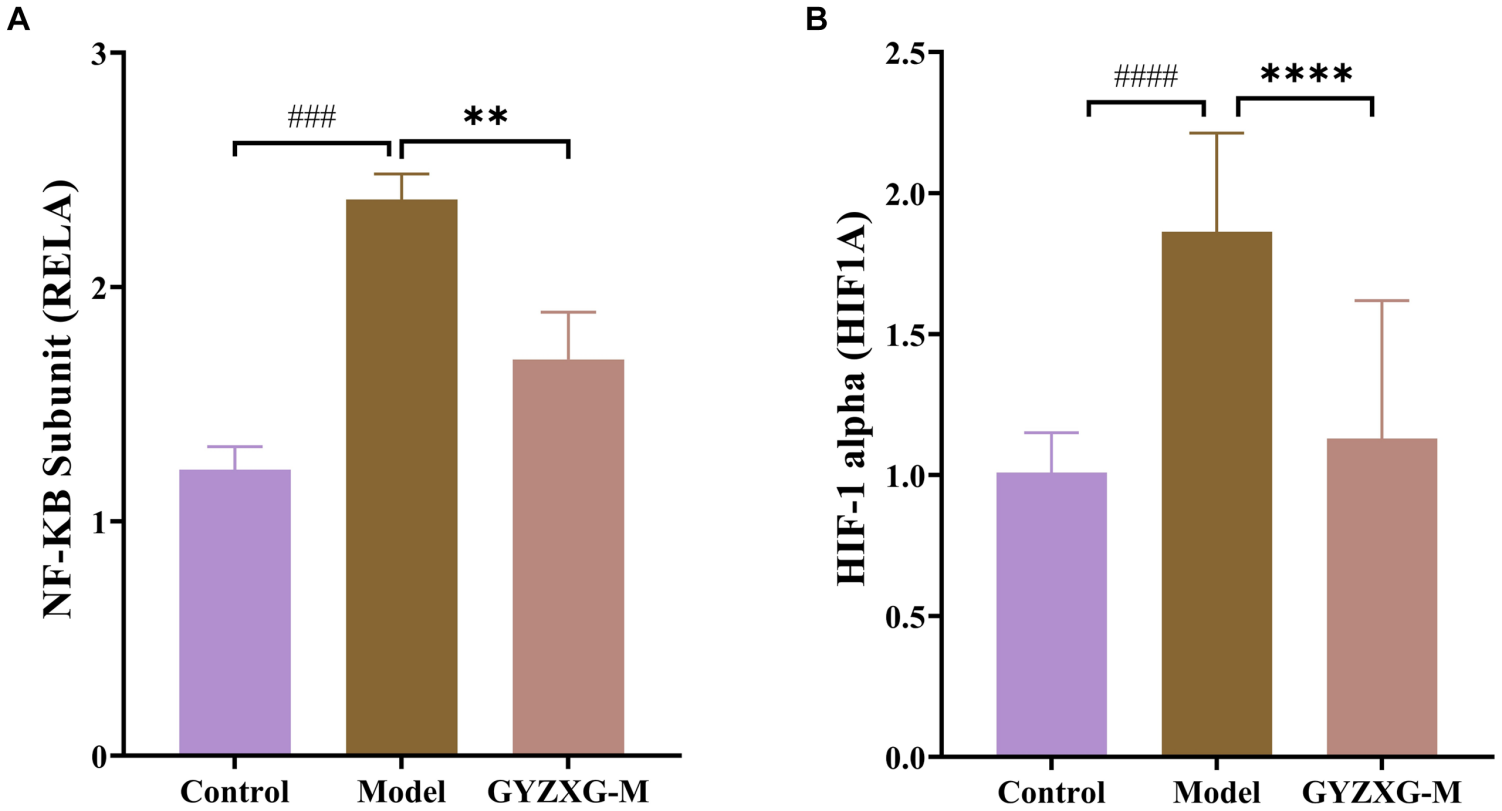
mRNA levels of **(A)** *Rela*, **(B)** *Hif1a* in rat gastric tissue (*n* = 5). The values are the means ± SEMs. ^#^*p* < 0.05, ^##^*p* < 0.01 vs. the control group; ^*^p < 0.05, ^**^p < 0.01 vs. the model group.

According to the results of the enrichment analysis, the main anti-GU pathways influenced by GYZXG include the *HIF-1*, *PI3K-Akt*, *TNF* and apoptosis signaling pathways. Notably, *HIF-1* signaling is closely related to both the *PI3K-Akt* and Apoptosis signaling pathway. Therefore, we conducted RT–qPCR to validate the expression of genes related to *HIF-1* signaling. The RT–qPCR experiments showed that GYZXG significantly reduced the mRNA expression levels of *Rela* and *Hif1a* (*p* < 0.01). Conversely, the expression level of *Egf* mRNA in rat gastric tissue showed an increase, though this change was not statistically significant (*p* > 0.05; Figure 6).

### 4.8 Identification of characteristic serum metabolites and analysis of related metabolic pathways in GU rats

**Figure 7:**
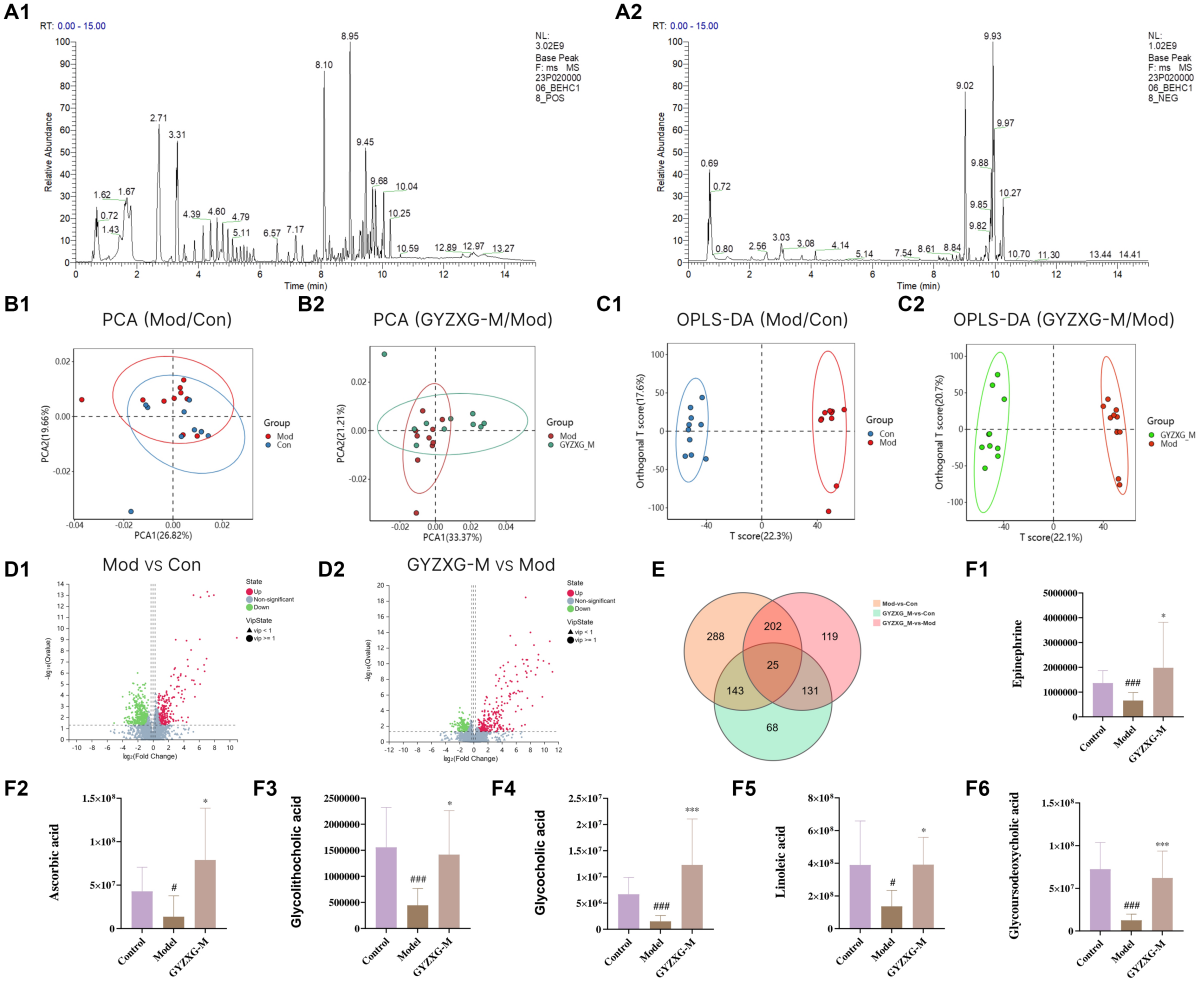
Metabolites of GYZXG protection and treatment against GU rats analyzed by untargeted metabolic profiling. **(A1,A2)** Total ion diagram in positive and negative ion modes. **(B1,B2)** Principal compound analysis score chart. **(C1,C2)** OPLS-DA scoring chart. **(D1,D2)** Metabolite volcano map. **(E)** Venn plot of differential metabolites. **(F1–F6)** Changes in differential metabolites. ^*^p < 0.05, ^**^p < 0.01, ^***^p < 0.001.

The metabolic profiles of the normal control group, model group, and GYZXG-M group were significantly separated in both positive and negative ion modes, as demonstrated by the principal compound analysis (PCA) score plots (Figures 7A1,A2,B1,B2), indicating significant differences between the groups. OPLS-DA analysis further characterized these metabolic differences. As depicted in Figures 7C1,C2, the score plots for each group exhibited distinct patterns, with each group clustering separately. Each group was clustered separately, indicating that these three groups were different and demonstrated good reproducibility.

**Figure 8:**
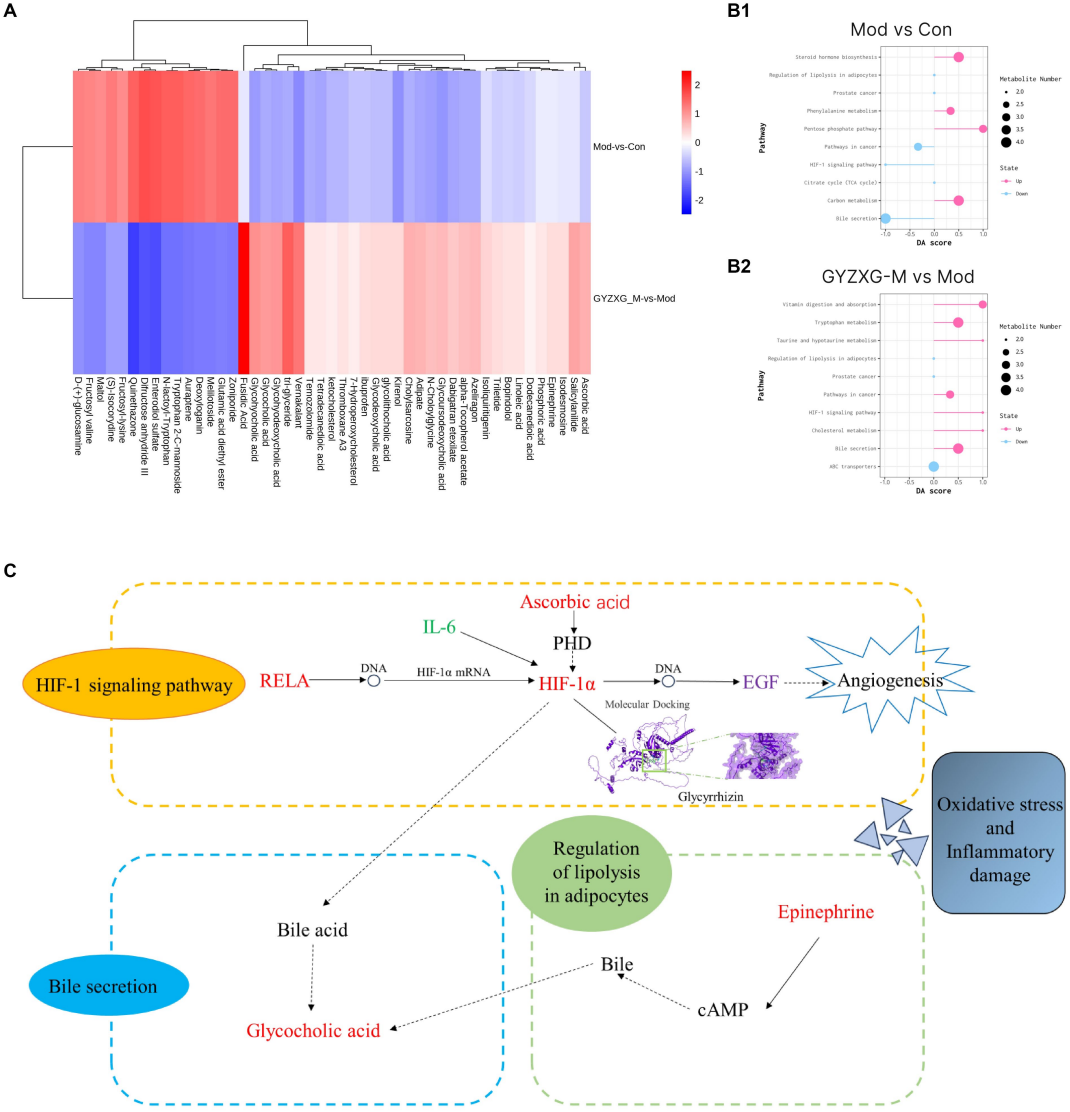
**(A)** Heat map of 47 differential metabolites. **(B1,B2)** Analysis of differential metabolite metabolism pathways in GU rats. **(C)** The main mechanism of action of GYZXG on GU rats.

Following the OPLS-DA model, we screened potential differential metabolites in both positive and negative ion modes based on VIP > 1.0 and *p* < 0.05 (Figures 7D1,D2). The results indicated that 658, 477, and 227 differential metabolites were identified in the model group compared to the normal control group, the GYZXG-M group compared to the model group, and the intersection group, respectively (Figure 7E). Using additional criteria of FC > 1.2 or < 0.83, we found that of the 524 differential metabolites in the model group versus the normal control group, 121 exhibited a substantial increase, while 403 showed a significant decrease. Among the 385 differential metabolites in the GYZXG-M group compared to the model group, 198 increased notably, and 187 decreased significantly. Nonetheless, initial annotations were performed in the intersection group consisting of 185 differentially expressed metabolites utilizing the KEGG and HMDB database, identifying 47 key differentially expressed metabolites (Appendix Table). Subsequently, through further analysis of the data on these 47 differential metabolites in the intersection group and comparison with the model group, it was found that 32 differential metabolites showed a significant up-regulation, while 15 differential metabolites experienced a notable down-regulation in the GYZXG-M group. Adjusting the levels of these differential metabolites helped restore them to a normal range, indicating a strong correlation between these 47 metabolites and the intervention on GU by GYZXG (Figure 8A). Notably, Epinephrine, Ascorbic acid, Glycolithocholic acid, Glycocholic acid, Linoleic acid, and Glycoursodeoxycholic acid, among others, were significantly lower than normal levels in the model group (*p* < 0.05); However, the administration of GYZXG could effectively restore these metabolites to near-normal levels (*p* < 0.05; Figures 7F1–F6).

Then, the most relevant metabolic pathways of GYZXG in intervening in anhydrous ethanol-induced GU in rats were explored based on differential metabolites. The experiment revealed significant changes in the Bile secretion metabolism, *HIF-1* signaling pathway, and Adipocyte breakdown metabolism pathway in the rats of the model group compared to the normal control group (*p* < 0.01; Figure 8B1). On the other hand, the GYZXG-M group restored these three metabolic pathways and effectively intervened in GUs when compared to the model group (*p* < 0.01; Figures 8B2,C).

### 4.9 GYZXG can significantly improve the gut microbiota structure of GU rats

**Figure 9:**
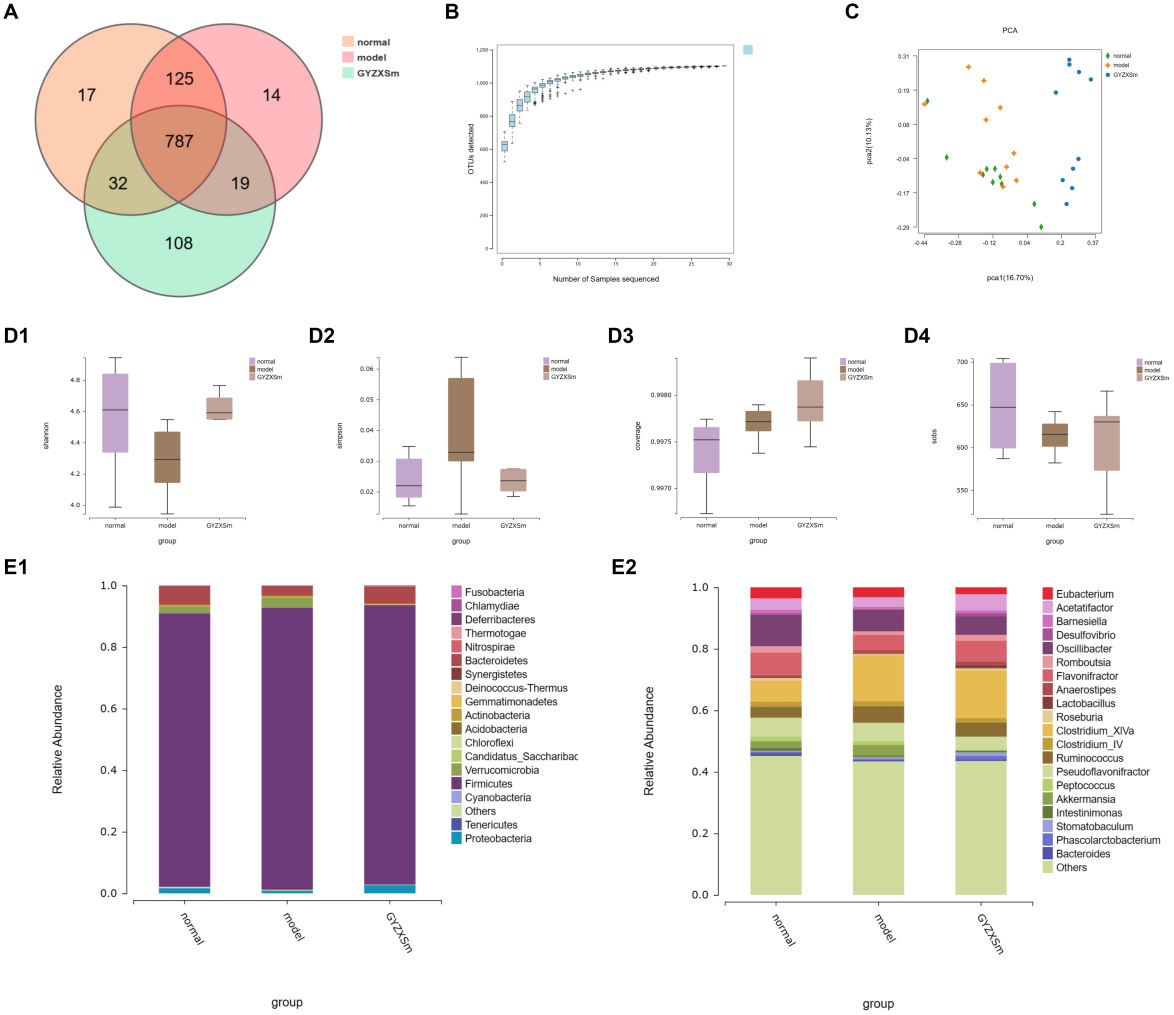
Results of the diversity analysis of gut microbial communities in the three groups. **(A)** OTU-based Venn diagram for three groups. **(B)** Sparse curve. **(C)** Score plot of PCA analysis of gut microbiota. **(D1–D4)** Box plots of intergroup differences in Alpha diversity. **(E1,E2)** Gut microbial community abundance at phylum and genus level.

Using 16S rRNA high-throughput sequencing technology, we analyzed the cecal contents of rats from normal control, model, and GYZXG-M group. We identified a total of 787 abundances from 1,102 OTUs across the three groups (Figure 9A). Additionally, as the volume of sequenced samples increased, the corresponding rarefaction curve approached saturation, indicating that the number of species did not increase linearly or rapidly. This suggests that the sequencing data were sufficient and reliable (Figure 9B).

Compared with the normal control group, the Simpson index of the model group was observed to gradually increase, whereas the Simpson index of the GYZXG-M intervention group showed a decreasing trend. This suggests that establishing a GU model leads to increased gut microbiota diversity, while the GYZXG-M intervention could restore this diversity. In comparison to the normal control group, the model group exhibited a decline in the Shannon, Coverage, and Sobs indices, signifying reduced gut microbiota diversity following the modeling process. Conversely, the Shannon, Coverage, and Sobs indices in the GYZXG-M intervention group demonstrated an ascending pattern compared to the model group (Figures 9D1–D4). These findings indicate that the gut microbiota diversity in the GYZXG-M intervention group is approaching that of the normal control group.

Beta diversity analysis (OTU PCA results) showed that PCA1 and PCA2 accounted for 16.70 and 10.13% of the sample differences, respectively, indicating significant differences among the three groups of samples. Notably, the GYZXG-M group was positioned far from the model group and appeared to be closer to the normal control group (Figure 9C). These results suggest that GYZXG partially restored the richness and diversity of the gut microbiota in GU rats, although its impact on the β-diversity index of the gut microbiota was limited.

**Figure 10:**
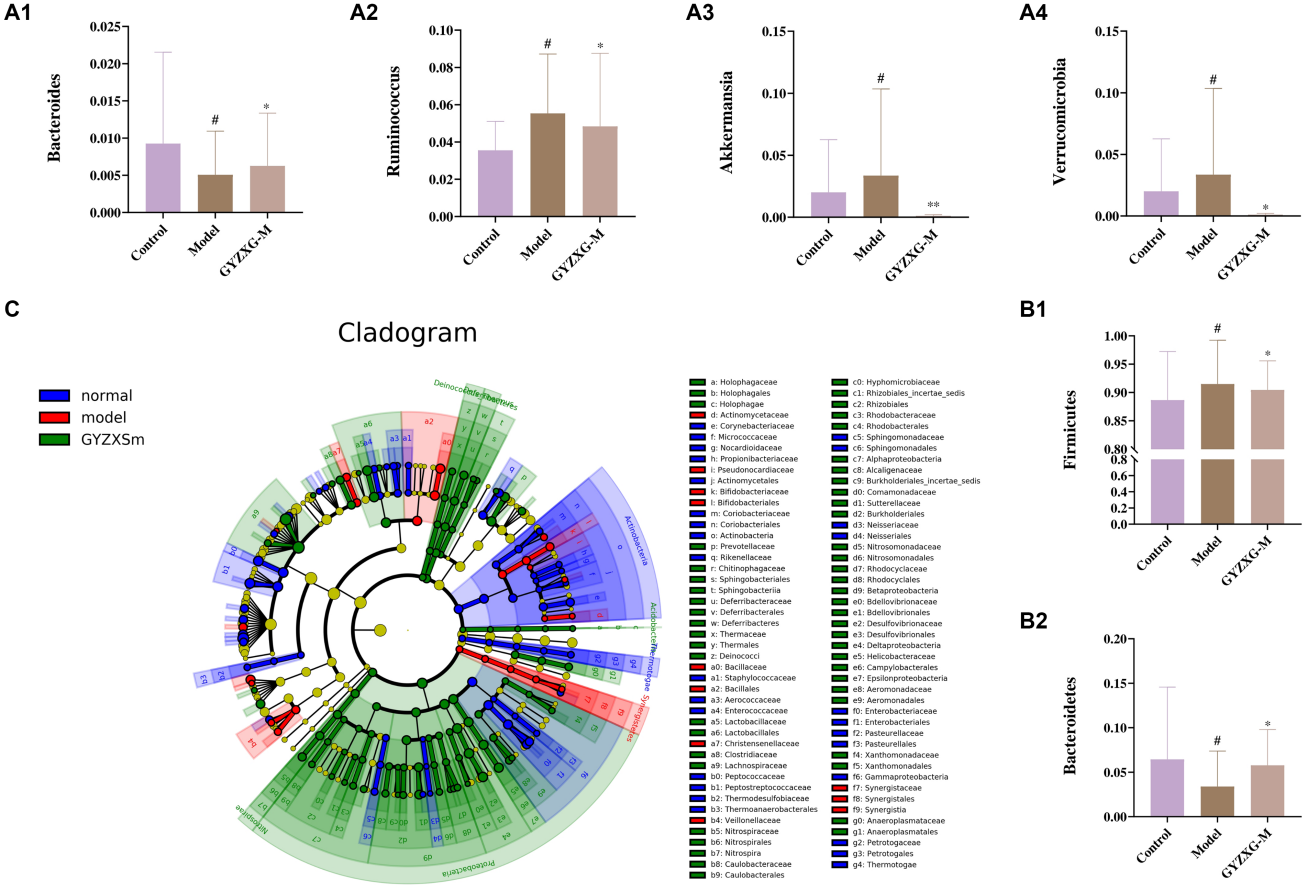
**(A1–A4,B1,B2)** Effect of GYZXG on the relative abundance of gut microbiota at genus and phylum level. **(C)** LEfSe analysis of gut microbiota. ^*^p < 0.05, ^**^p < 0.01, ^***^p < 0.001.

The gut flora was analyzed structurally, and at the phylum level, the five most abundant species were *Firmicutes*, *Bacteroidetes*, *Verrucomicrobia*, *Proteobacteria*, and *Actinobacteria*, which accounted for the majority of all taxa (Figure 9E1). Subsequently, differences in microbial communities at the genus level were compared among these taxa. Seven genera showed significant differences between the normal control group and the model group (Figure 9E2; *p* < 0.05). In the normal control group, the relative abundances of *Clostridium_XlVa*, *Bacteroides*, *Ruminococcus*, and *Barnesiella* were significantly higher, whereas they were significantly lower in the model group (*p* < 0.05). After GYZXG treatment, the relative abundance of these phyla and genera approached near-normal levels compared to the model group (Figures 10A1–A4,B1,B2).

To determine the specific microbial community associated with GYZXG in the intervention on GU caused by anhydrous ethanol, LEfSe analysis was used to compare changes in bacterial phyla and genera among the three groups. Significant differences were observed in the composition of the gut microbiota across these groups. The results indicated that *Firmicutes*, *Clostridiales*, *Ruminococcaceae*, and *Oscillibacter* were the most abundant in the model group, with LDA scores (Log10) > 4. To determine the specific microbial community associated with GYZXG in the intervention on GU caused by anhydrous ethanol, LEfSe analysis was used to compare changes in bacterial phyla and genera among the three groups. Significant differences were observed in the composition of the gut microbiota across these groups. The results indicated that *Firmicutes*, *Clostridiales*, *Ruminococcaceae*, and *Oscillibacter* were the most abundant in the model group, with LDA scores (Log10) of >4, suggesting a reshaping of the intestinal microbiota to some extent (Figure 10C).

### 4.10 Spearman correlation analysis between serum differential metabolites and differential bacterial communities

**Figure 11:**
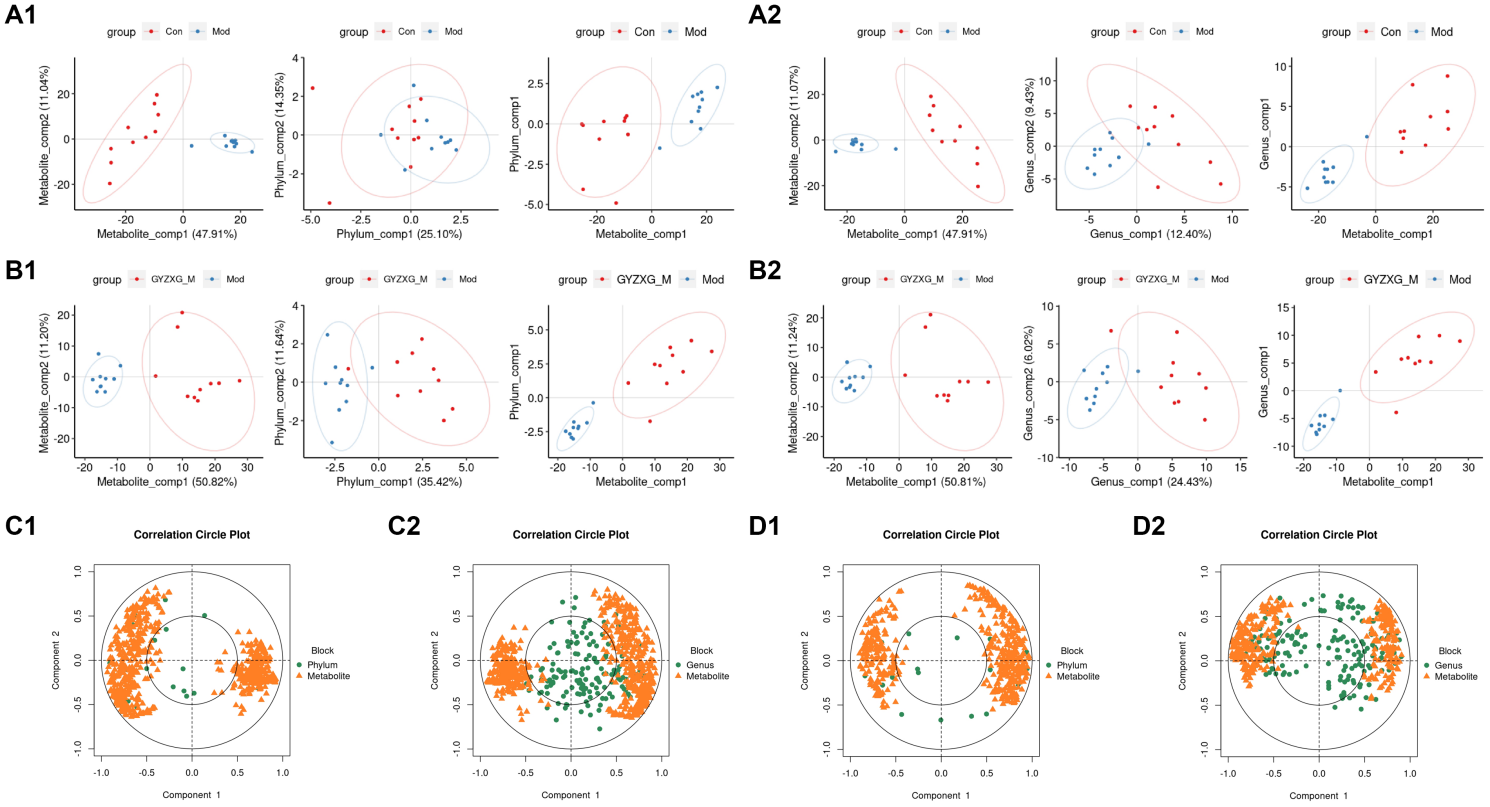
**(A1,A2,B1,B2)** Phylum and genus level principal compound analysis score chart. **(C1,C2,D1,D2)** Correlation coefficients between phylum level and genus level and serum metabolic phenotypes.

Finally, the potential relationship between the gut microbiota, analyzed through 16S rRNA gene sequencing, and serum metabolites, analyzed through serum metabolomics, was further explored. Spearman correlation analysis was conducted on the differential flora and metabolites (Figures 11A1,A2,B1,B2). Figures 11C1,C2,D1,D2 illustrate the correlation coefficient between the relative abundance of highly correlated gut microbiota and serum metabolic phenotypes in the model and GYZXG-M groups. The results revealed that metabolites down-regulated in the model group were up-regulated following GYZXG intervention. At the phylum level, the abundance of *Actinobacteria* was negatively correlated with Glycocholic acid (*r* = −0.496), while the abundance of *Bacteroidetes* was positively correlated with Epinephrine (*r* = 0.457). Moreover, the abundance of *Verrucomicrobia* was negatively correlated with Glycocholic acid (*r* = −0.501), and *Proteobacteria* showed positive correlations with Epinephrine (*r* = 0.749), Ascorbic acid (*r* = 0.550), Glycolithocholic acid (*r* = 0.507), and Glycocholic acid (*r* = 0.660). At the genus level, the abundance of *Barnesiella* was positively correlated with Epinephrine (*r* = 0.437), and the abundance of *Akkermansia* was negatively correlated with Glycolic acid (*r* = −0.501). These results suggest that GYZXG’s improvement of GU is closely related to changes in gut microbiota and serum metabolites.

## 5 Discussion

The incidence of gastrointestinal ulcerative diseases has significantly increased with the rapid development of intensive livestock and poultry farming modes. Chinese medicine compounds have broad application prospects because they do not easily induce bacterial resistance, are multi-targeted, environmentally friendly, and can enhance the immunity of livestock and poultry (22). In this study, we first used the plant untargeted metabolism technique to analyze and identify the active compounds of GYZXG. Combined with network pharmacological predictions and metabolomics analysis, it was revealed that the *HIF-1* signaling pathway is closely associated with the anti-GU effects of GYZXG. We established a GU disease model in rats by gavage with anhydrous ethanol and constructed a classic GU injury model (23, 24). Research has shown that stimulation with anhydrous ethanol (1 mL/200 g) leads to severe pathological changes in the gastric mucosa of rats, confirming that the construction of the GU model in this experiment was consistent with previous reports (15, 25, 26). The results of this study indicated that the intervention in anhydrous ethanol-induced GU with GYZXG was associated with changes in the levels of inflammatory and growth factors. Specifically, the levels of pro-inflammatory factors *IL-6* and *TNF-α* significantly increased, while the levels of anti-inflammatory factors *IL-10* and *IL-4* were significantly reduced. Similarly, the content of *PP* increased significantly, whereas the content of *EGF* decreased significantly. Furthermore, after 1 week of prophylactic administration of GYZXG, the degree of gastric injury decreased, particularly in the medium-dose group, where the ulcer inhibition rate reached nearly 50%. Additionally, the levels of *IL-6*, *TNF-α*, and *PP* decreased to varying degrees, and the levels of *IL-10*, *IL-4*, and *EGF* increased correspondingly. Metabolomics and 16S rRNA analysis showed that GYZXG restored relevant metabolites and gut microbiota in GU rats induced by anhydrous ethanol. Spearman’s analysis indicated that gut microbiota disorders were strongly associated with changes in several relevant metabolites, suggesting that metabolites related to gut microbiota under GYZXG intervention may improve GU by regulating host tissue function (27). These findings suggest that the mechanism by which GYZXG intervenes in GU in rats may involve regulating the gut microbiota and its metabolites.

Firstly, to explore the material foundation for GYZXG’s intervention on GU in rats, the active compounds of GYZXG were analyzed using the UHPLC-Q/TOF-MS technique. A total of 33 active compounds were identified, including Flavonoids, Triterpenoids, Lipids, Tannins, Alkaloids, and others. Combined with network pharmacological prediction results, the mass spectra and 2D structures for nine of these compounds—closely related to GYZXG—were analyzed, including Quercetin, Naringenin, Licochalcone A, Formononetin, Luteolin, Kaempferol, Matrine, Gallic acid, Glycyrrhizic acid. Shams et al. (28) found that Quercetin significantly reduced gastric volume by 86% and gastric lesion count by 3.5 times in an ethanol-induced GU rat model, attributing these effects to its antioxidant, anti-inflammatory, and anti-apoptotic activities. Emin et al. (29) reported that naringenin ameliorated histopathological abnormalities in the stomach and provided protection by decreasing levels of *TNF-α*, *IL-6*, *CRP*, *iNOS*, and *Caspase-3*, and by increasing *COX-2* levels in a rat model of indomethacin-induced GUs. Licochalcone A has anti-ulcer, antibacterial, anti-inflammatory properties, anti-inflammatory properties, and significant free radical scavenging and antioxidant effects (30, 31). Yi et al. (32) investigated the effects and mechanisms of Formononetin on GUs in rats, clarifying that Formononetin could improve GUs by inhibiting inflammation and promoting gastric mucosal angiogenesis. Wang et al. (33) explored the protective effects and mechanisms of Luteolin in preventing and treating GUs induced by indomethacin in rats, showing that Luteolin reduced GUs dose-dependently and improved pathological changes in the gastric mucosa. Jia et al. (34) conducted a comparative study on the pharmacokinetics of five flavonoids in the treatment of GUs in rats using Mongolian medicine Shudage-4 through UPLC-ESI-MS/MS. The results showed that the metabolism and transport of compounds such as Kaempferol were faster in GU rats than in normal rats, suggesting that these compounds may accumulate and exert effects at the site of disease progression. Yamazaki confirmed through pharmacological research that Matrine has a protective effect on restraint and immersion-induced stress ulcers in mice and could inhibit ulcer formation (35). Zhou et al. (36) explored the potential mechanism of action of Gallic acid on anhydrous ethanol-induced GUs in rats, revealing that its preventive and therapeutic effects might be related to its anti-apoptotic and antioxidant properties. Glycyrrhizic acid, a triterpenoid saponin, has antioxidant, anti-inflammatory, hepatoprotective, and detoxification effects, effectively alleviating inflammatory responses and immune stress in livestock and poultry. It also has a potent inhibitory effect on various experimental GUs at certain doses (37, 38). Among these, Matrine, Gallic acid, and Glycyrrhizic acid were identified as individual compounds, consistent with previous results obtained by thin-layer chromatography and high-performance liquid chromatography by our research group (10). This provides a reference for further research into the active compounds and mechanisms of action of drugs, offering a theoretical basis for exploring the intervention effects of GYZXG on GU in animal experiments.

Secondly, the serum metabolomics analysis of each group of rats revealed differential metabolites including Epinephrine, Ascorbic acid, Glycolithocholic acid, Glycocholic acid, Linoleic acid, and Glycoursodeoxycholic acid. Simultaneously, critical metabolic pathways identified were Bile secretion metabolism, *HIF-1* signaling pathway, and Adipocyte catabolism. Glycocholic acid a major component of animal bile, is known for its anti-inflammatory properties, which can significantly inhibit the secretion of pro-inflammatory cytokines and the production of macrophages. Conversely, an increase in Glycocholic acid levels can inhibit excessive bile acid production, exacerbating gastric ulcerative lesions by promoting bile reflux into the stomach and damaging the gastric mucous barrier (39, 40). Epinephrine, a hormone produced by the adrenal gland, is released into the bloodstream during stress and promotes vasoconstriction, reducing gastric mucosal blood flow and thereby diminishing bleeding and inflammation in ulcers. Yildirim et al. (41) found that adrenalectomy in rats altered the levels of antioxidants and lipid peroxidation products in gastric tissues and erythrocytes, whereas epinephrine injections maintained oxidative/antioxidant homeostasis. Ascorbic acid, a potent antioxidant, scavenges free radicals, reduces inflammation, and protects cells from oxidative damage (42). Koc et al. (43) found that ascorbic acid reduced the occurrence of indomethacin-induced gastric injury, possibly by modulating the antioxidant system and myeloperoxidase activity. Furthermore, the study found that Ascorbic acid levels increased in rats with anhydrous ethanol-induced GU and negatively correlated with *IL-6*, inhibiting the production and release of *IL-6*, reducing the production of pro-inflammatory cytokines, and helping to decrease chronic inflammation and the immune response (44). Research has also shown that while ascorbic acid does not promote growth, its interaction with *EGF* can enhance *EGF* production, thereby promoting cell repair and regeneration (45). In this study, the Ascorbic acid content in metabolites of the GYZXG-M group rats was significantly increased, *IL-6* levels were significantly decreased, and the *EGF* levels were significantly increased. It is suggested that the changes in metabolite ascorbic acid in the GYZXG intervention of GUs are related to oxidative stress and inflammatory response. The intervention of GYZXG restored abnormal changes in Ascorbic acid, *IL-6*, and *EGF* in GU rats, suggesting that the regulatory effect of GYZXG on Ascorbic acid was related to GU injury. This further suggests that GYZXG may play a protective role by restoring oxidative stress and inflammatory damage caused by the *HIF-1* signaling pathway metabolism on the gastric mucosa.

An interaction network between GYZXG active compounds, corresponding targets, and GU-related genes was established early on through network pharmacology analysis (46). The results of the analysis indicated that the pathway of GYZXG active compounds in intervening in GU in rats was closely related to the *HIF-1* signaling pathway. RT-qPCR was utilized to validate the key genes of the *HIF-1* signaling pathway, and it was elucidated that within the *HIF-1* signaling pathway, GYZXG was involved in regulating angiogenesis to intervene in GU by modulating the key targets *RELA*, *HIF1A*, and *EGF*. Finally, the key targets were subjected to a reverse search for the components that interact with them, and molecular docking validation was carried out. This study found that the *HIF-1* signaling pathway may be the focal point of network pharmacology, metabolomics pathways, and the metabolite Ascorbic acid, further confirming that GYZXG may intervene in GUs by regulating the *HIF-1* signaling pathway and its associated metabolites.

In this study, significant changes were observed in the diversity of gut microbiota in rats following the onset of GU. Under the intervention of GYZXG, the gut microbiota showed a tendency to recover. After exploring the differential metabolites and metabolic pathway changes induced by GYZXG in rats with anhydrous ethanol-induced GUs, the further research focused on the relationship between gut microbiota and GYZXG intervention in GU. Some studies have shown that consuming traditional Chinese medicine preparations could alter the distribution of gut microbiota (27, 47). During the development of GU, multiple microbiotas are involved, and the relative abundance of *Firmicutes*, *Bacteroidetes*, *Verrucomicrobia*, *Proteobacteria*, *Actinobacteria*, *Ruminococcus*, and other microbiota directly affects the intervention on GUs (48, 49). According to previous studies (50, 51), significant changes in gut microbiota abundance were observed at the phylum and genus levels in our study. Compared to the model group, the relative abundance of these phyla and genus was adjusted to near-normal levels. Notably, *Bacteroidetes*, a beneficial bacterium, is involved in essential metabolic activities and has been found to attenuate atherosclerotic lesions, markedly ameliorate endotoxemia, reduce the production of intestinal microbial lipopolysaccharides, and effectively inhibit pro-inflammatory immune responses (52). In this study, *Bacteroidetes* at the phylum level and *Bacteroides* at the genus level were down—regulated in the GU rat model group and up-regulated in the GYZXG—administered group. An increase in the relative abundance of *Bacteroides* can regulate Bile secretion metabolism, reduce cell apoptosis (53), and participate in the metabolism and absorption of drug-active compounds by regulating the *HIF-1* signaling pathway (54). *Verrucomicrobia*, which is abundant in the inner layer of the intestinal mucosa of healthy individuals, plays a crucial role in regulating intestinal health and the immune system. Lindenberg et al. (55) explored the relationship between the expression of immunomodulatory genes and the abundance of *Verrucomicrobia*, indicating its potential to induce regulatory immunity and identifying it as a possible target for microbial interventions aimed at improving regulatory immunity. *Akkermansia*, a bacterium within the *Verrucomicrobia* phylum, is known to reduce inflammation and regulate immune responses. Lim et al. (56) found that the number of *Akkermansia* significantly increased in the treatment group of GU patients. *Firmicutes*, a common gut microbiota, often exist as beneficial bacteria. In this study, compared with the GU model group, the relative abundance of *Firmicutes* in the GYZXG intervention group decreased. Still, the number of *Ruminococcus* in the genus level significantly increased compared to the GU model group. As a member of *Firmicutes*, *Ruminococcus* can stabilize the intestinal barrier, increase energy, and decompose complex substances to reduce gastric damage. Huang et al. (57) demonstrated that small black soybean (*Vigna Mungo* L.) polysaccharides effectively ameliorated intestinal microbiota dysbiosis in GU rats. Their study highlighted the dual role of these polysaccharides in treating GUs by reducing *Firmicutes* levels and increasing *Ruminococcus* levels.

## 6 Conclusion

The results of the present study confirm the protective effects of GYZXG against anhydrous ethanol-induced GU in rats. GYZXG intervenes in GU by restoring gut microbiota dysbiosis, modulating Glycocholic acid, Ascorbic acid, and Bile secretion metabolism, as well as the *HIF-1* signaling pathway. It also regulates inflammatory factors, pepsin (*PP*), and epidermal growth factor (*EGF*). Additionally, changes in key genes of the *HIF-1* signaling pathway—*RELA*, *HIF1A*, and *EGF*—detected through PT-qPCR, which affect Angiogenesis, further illustrate the mechanism of GYZXG’s action in intervening GU. The joint analysis of gut microbiota and metabolites offers a new perspective for exploring the intervention mechanisms of GYZXG on GUs.

## Data availability statement

The data presented in the study are deposited in the https://www.ncbi.nlm.nih.gov/, https://www.ebi.ac.uk/metabolights/ repository, accession numbers PRJNA1103996; MTBLS10026.

## Ethics statement

The animal study was approved by Animal Ethics Committee of Gansu Agricultural University. The study was conducted in accordance with the local legislation and institutional requirements.

## Author contributions

TM: Conceptualization, Formal analysis, Methodology, Software, Validation, Writing – original draft. PJ: Conceptualization, Funding acquisition, Project administration, Supervision, Writing – review & editing. F-LW: Project administration, Supervision, Writing – review & editing. C-CL: Data curation, Investigation, Writing – review & editing. J-QD: Investigation, Software, Visualization, Writing – review & editing. H-CY: Data curation, Investigation, Software, Writing – review & editing. Y-MW: Funding acquisition, Supervision, Writing – review & editing. Y-LH: Project administration, Supervision, Visualization, Writing – review & editing.

## Footnotes

^1^ http://tcmspw.com/tcmsp.php

^2^ http://www.ncbi.nlm.nih.gov/geo/

^3^ https://www.genecards.org/

^4^ https://omim.org/

^5^ https://www.pharmgkb.org/

^6^ http://db.idrblab.net/ttd/

^7^ https://www.drugbank.ca/

^8^ http://stringdb.org/

^9^ https://pubchem.ncbi.nlm.nih.gov/

^10^ http://www.rcsb.org/

^11^ http://www.genome.jp/kegg/

^12^ https://hmdb.ca/

^13^ https://old.tcmsp-e.com/tcmsp.php

## Appendix Table

Information on 47 key differential metabolites

**Table:**

No.	Metabolites	Formula	Mod/Con	Trend	P		GYZXGm/Mod	Trend	P	
			VIP	FC			VIP	FC		
1	Difructose anhydride III	C_12_H_20_O_10_	1.899	2.28	↑	**	2.335	-2.57	↓	**
2	Enterodiol sulfate	C_18_H_22_O_7_S	1.955	2.17	↑	***	2.402	-2.69	↓	**
3	N-lactoyl-Tryptophan	C_14_H_16_N_2_O_4_	1.911	2.08	↑	***	2.022	-2.03	↓	***
4	Tryptophan2-C-mannoside	C_17_H_22_N_2_O_7_	1.884	2.06	↑	***	2.002	-2.02	↓	***
5	Quinethazone	C_10_H_12_C_l_N_3_O_3_S	1.623	1.94	↑	*	2.140	-3.01	↓	**
6	Auraptene	C_19_H_22_O_3_	1.822	1.84	↑	***	1.945	-1.83	↓	***
7	Deoxyloganin	C_17_H_26_O_9_	1.589	1.80	↑	**	1.805	-1.78	↓	*
8	Glutamic acid diethyl ester	C_9_H_17_NO_4_	1.728	1.74	↑	***	1.781	-1.65	↓	**
9	Zoniporide	C_17_H_16_N_6_O	1.795	1.74	↑	***	1.910	-1.76	↓	**
10	Melilotoside	C_15_H_18_O_8_	1.568	1.61	↑	**	1.804	-1.73	↓	**
11	(S)-Isocorydine	C_20_H_23_NO_4_	1.519	1.56	↑	**	1.091	-1.05	↓	*
12	D-(+)-glucosamine	C_6_H_13_NO_5_	1.502	1.47	↑	**	1.612	-1.39	↓	*
13	Fructosyl valine	C_11_H_21_NO_7_	1.488	1.36	↑	**	1.816	-1.58	↓	**
14	Maltol	C_6_H_6_O_3_	1.480	1.27	↑	***	1.771	-1.57	↓	**
15	Fructosyl-lysine	C_12_H_24_N_2_O_7_	1.391	1.26	↑	**	1.394	-1.06	↓	*
16	Phosphoric acid	H_3_O_4_P	1.230	-1.01	↓	**	1.454	1.46	↑	*
17	Epinephrine	C_9_H_13_NO_3_	1.308	-1.05	↓	**	1.420	1.59	↑	*
18	Fusidic Acid	C_31_H_48_O_6_	1.275	-1.09	↓	*	3.591	6.22	↑	***
19	Salicylanilide	C_13_H_11_NO_2_	1.295	-1.12	↓	**	2.328	2.79	↑	***
20	Isodesmosine	C_24_H_40_N_5_O_8_	1.351	-1.14	↓	*	1.475	1.62	↑	*
21	Isoliquiritigenin	C_15_H_12_O_4_	1.277	-1.30	↓	*	1.448	1.84	↑	*
22	Dodecanedioic acid	C_12_H_22_O_4_	1.280	-1.32	↓	*	1.190	1.25	↑	*
23	Bopindolol	C_23_H_28_N_2_O_3_	1.636	-1.46	↓	***	1.731	1.55	↑	***
24	Linoleic acid	C_18_H_32_O_2_	1.540	-1.52	↓	*	1.709	1.52	↑	*
25	Triletide	C_27_H_31_N_5_O_5_	1.797	-1.55	↓	**	1.691	1.60	↑	**
26	Ascorbic acid	C_6_H_8_O_6_	1.944	-1.64	↓	*	2.146	2.53	↑	**
27	ibuprofen	C_13_H_18_O_2_	1.826	-1.68	↓	***	1.775	1.57	↑	***
28	7-Hydroperoxycholesterol	C_27_H_46_O_3_	1.869	-1.72	↓	***	1.484	1.29	↑	**
29	Glycodeoxycholic acid	C_26_H_43_NO_5_	1.950	-1.81	↓	**	1.738	1.72	↑	**
30	glycolithocholic acid	C_26_H_43_NO_4_	1.886	-1.81	↓	**	1.622	1.67	↑	*
31	ketocholesterol	C_27_H_44_O_2_	1.981	-1.97	↓	***	1.533	1.34	↑	**
32	Thromboxane A3	C_20_H_30_O_5_	1.612	-2.00	↓	*	1.560	1.51	↑	*
33	Cholylsarcosine	C_27_H_45_NO_6_	1.607	-2.04	↓	*	1.765	2.60	↑	*
34	alpha-Tocopherol acetate	C_31_H_52_O_3_	2.093	-2.11	↓	***	1.841	2.22	↑	*
35	tri-glyceride	C_9_H_12_O_9_	1.942	-2.14	↓	*	2.408	4.24	↑	*
36	Glycocholic acid	C_26_H_43_NO_6_	1.932	-2.14	↓	***	2.365	3.02	↑	***
37	Azeliragon	C_32_H_38_C_l_N_3_O_2_	1.986	-2.19	↓	***	1.526	2.33	↑	*
38	Temozolomide	C_6_H_6_N_6_O_2_	2.125	-2.19	↓	***	1.456	1.46	↑	*
39	Tetradecanedioic acid	C_14_H_26_O_4_	1.952	-2.22	↓	***	1.400	1.46	↑	*
40	Dabigatran etexilate	C_34_H_41_N_7_O_5_	2.089	-2.23	↓	***	1.998	2.04	↑	***
41	Adipate	C_6_H_10_O_4_	1.808	-2.27	↓	**	1.887	2.51	↑	*
42	Glycohyodeoxycholic acid	C_26_H_43_NO_5_	2.024	-2.27	↓	***	2.211	3.11	↑	**
43	N-Choloylglycine	C_26_H_43_NO_6_	2.167	-2.37	↓	***	1.920	2.30	↑	**
44	Glycohyocholic acid	C_26_H_43_NO_6_	2.145	-2.45	↓	***	2.454	3.24	↑	***
45	Glycoursodeoxycholic acid	C_26_H_43_NO_5_	2.228	-2.53	↓	***	1.987	2.31	↑	**
46	Kirenol	C_20_H_34_O_4_	2.185	-2.56	↓	***	1.522	1.71	↑	*
47	Vernakalant	C_20_H_31_NO_4_	1.726	-2.69	↓	*	2.297	3.76	↑	*
